# N4-acetylcytidine regulates the replication and pathogenicity of enterovirus 71

**DOI:** 10.1093/nar/gkac675

**Published:** 2022-08-16

**Authors:** Haojie Hao, Weichi Liu, Yuanjiu Miao, Li Ma, Baocheng Yu, Lishi Liu, Chunjie Yang, Kui Zhang, Zhen Chen, Jingwen Yang, Zhenhua Zheng, Bo Zhang, Fei Deng, Peng Gong, Jianhui Yuan, Zhangli Hu, Wuxiang Guan

**Affiliations:** College of Life Sciences and Oceanography, Shenzhen University, Shenzhen 518060, China; College of Physics and Optoelectronic Engineering, Shenzhen University, Shenzhen 518060, China; Hanshan Normal University, Chaozhou 521041, China; Center for Emerging Infectious Diseases, Wuhan Institute of Virology, Chinese Academy of Sciences, Wuhan, Hubei 430071, China; Center for Emerging Infectious Diseases, Wuhan Institute of Virology, Chinese Academy of Sciences, Wuhan, Hubei 430071, China; Center for Emerging Infectious Diseases, Wuhan Institute of Virology, Chinese Academy of Sciences, Wuhan, Hubei 430071, China; University of Chinese Academy of Sciences, Beijing 100049, China; Center for Emerging Infectious Diseases, Wuhan Institute of Virology, Chinese Academy of Sciences, Wuhan, Hubei 430071, China; University of Chinese Academy of Sciences, Beijing 100049, China; Center for Emerging Infectious Diseases, Wuhan Institute of Virology, Chinese Academy of Sciences, Wuhan, Hubei 430071, China; University of Chinese Academy of Sciences, Beijing 100049, China; Center for Emerging Infectious Diseases, Wuhan Institute of Virology, Chinese Academy of Sciences, Wuhan, Hubei 430071, China; University of Chinese Academy of Sciences, Beijing 100049, China; Center for Emerging Infectious Diseases, Wuhan Institute of Virology, Chinese Academy of Sciences, Wuhan, Hubei 430071, China; Center for Emerging Infectious Diseases, Wuhan Institute of Virology, Chinese Academy of Sciences, Wuhan, Hubei 430071, China; University of Chinese Academy of Sciences, Beijing 100049, China; Center for Emerging Infectious Diseases, Wuhan Institute of Virology, Chinese Academy of Sciences, Wuhan, Hubei 430071, China; Center for Emerging Infectious Diseases, Wuhan Institute of Virology, Chinese Academy of Sciences, Wuhan, Hubei 430071, China; Center for Emerging Infectious Diseases, Wuhan Institute of Virology, Chinese Academy of Sciences, Wuhan, Hubei 430071, China; Center for Emerging Infectious Diseases, Wuhan Institute of Virology, Chinese Academy of Sciences, Wuhan, Hubei 430071, China; Center for Emerging Infectious Diseases, Wuhan Institute of Virology, Chinese Academy of Sciences, Wuhan, Hubei 430071, China; Center for Emerging Infectious Diseases, Wuhan Institute of Virology, Chinese Academy of Sciences, Wuhan, Hubei 430071, China; Nanshan District Center for Disease Control and Prevention, Shenzhen 518054, China; College of Life Sciences and Oceanography, Shenzhen University, Shenzhen 518060, China; Center for Emerging Infectious Diseases, Wuhan Institute of Virology, Chinese Academy of Sciences, Wuhan, Hubei 430071, China; Hubei JiangXia Laboratory, Wuhan, Hubei 430200, China

## Abstract

Chemical modifications are important for RNA function and metabolism. N4-acetylcytidine (ac4C) is critical for the translation and stability of mRNA. Although ac4C is found in RNA viruses, the detailed mechanisms through which ac4C affects viral replication are unclear. Here, we reported that the 5′ untranslated region of the enterovirus 71 (EV71) genome was ac4C modified by the host acetyltransferase NAT10. Inhibition of NAT10 and mutation of the ac4C sites within the internal ribosomal entry site (IRES) suppressed EV71 replication. ac4C enhanced viral RNA translation via selective recruitment of PCBP2 to the IRES and boosted RNA stability. Additionally, ac4C increased the binding of RNA-dependent RNA polymerase (3D) to viral RNA. Notably, ac4C-deficient mutant EV71 showed reduced pathogenicity *in vivo*. Our findings highlighted the essential role of ac4C in EV71 infection and provided insights into potential antiviral treatments.

## INTRODUCTION

Chemical modifications are critical for RNA metabolism and function, including RNA splicing (1), localization (2), transport (3), translation (4) and stability (5). Methylation modifications, particularly N6-methyladenosine (m6A) and 5-methylcytosine (m5C), have been reported to play important roles in regulating the functions of cellular and viral RNAs (6–8). However, few studies have examined N4-acetylcytidine (ac4C), the first and only acetylation event occurring on eukaryotic mRNAs (9,10).

Discovered in the 1960s (11), ac4C is conserved in all domains of life (12,13) and catalyzed by N-acetyltransferase 10 (NAT10) or a homologous enzyme in other species (14,15). In humans and yeast, ac4C is critical for the translation and stability of tRNA and for the biogenesis of rRNA (16–19). Recent studies using antibody-based RNA immunoprecipitation (RIP) followed by deep-sequencing (RIP-seq) have suggested that ac4C exists in mRNA and promotes the stability and translation efficiency of mRNA (20). In mRNA from NAT10/THUMPD1-overexpressing cells and in all known tRNA and rRNA substrates, ac4C deposition strictly requires the 5′-CCG-3′ motif (21–23). Moreover, NAT10 is highly conserved and associated with many diseases, including Hutchinson-Gilford progeria syndrome (24), gastric cancer (25), ovarian cancer (26), acute myeloid leukemia (27), colorectal cancer (28), and liver cancer (29), highlighting the pathological roles of ac4C in diseases.

Using mass spectrometry or RIP-seq with anti-ac4C antibodies, the ac4C modification has been detected in several RNA viruses, including Zika virus, Dengue virus, hepatitis C virus, poliovirus, human immunodeficiency virus type 1 (HIV-1) (30), and influenza A virus (IAV) (31). The formation of ac4C in HIV-1 RNA is catalyzed by NAT10, and this modification promotes viral replication by enhancing viral RNA stability (32). However, whether viruses replicating exclusively in the cytoplasm harbor ac4C and the effects of ac4C on viral cytoplasmic replication have not yet been elucidated.

As one of the major causative agents of hand-foot-and-mouth disease (33), enterovirus 71 (EV71) is a non-enveloped virus with a positive single-stranded RNA genome belonging to the *Enterovirus* genus within the *Picornaviridae* family (34). Its genome is approximately 7.4 kb long and includes a structured 5′ untranslated region (UTR), coding region, and 3′ UTR with a poly-A tail (35). Similar to other enteroviruses, EV71 does not have a 5′ end cap but contains an internal ribosome entry site (IRES), which harbors several stem-loops and can mediate the initiation of viral cap-independent protein synthesis by recruiting a number of IRES trans-acting factors (ITAFs). The polyprotein is translated from the EV71 genome and cleaved into 11 viral proteins. Among these proteins, 3D, the RNA-dependent RNA polymerase (RdRp) of EV71, can directly use genomic RNA as a template to synthesize negative-strand RNAs, followed by synthesis of a large number of positive-strand RNAs using the negative-strand RNAs.

In the current study, we characterized ac4C in the EV71 genome and examined the role of the host acetyltransferase NAT10 in EV71 infection and virus replication. The functions of ac4C in viral replication, protein expression, RNA stability, RNA binding, and viral pathogenesis were characterized. Our study revealed that the ac4C modification played important roles in EV71 infection.

## MATERIALS AND METHODS

### Cell culture

Vero (American Type Culture Collection [ATCC], Manassas, VA, USA; cat. no. CCL-81), RD (ATCC; cat. no. CCL-136), and HEK293T (ATCC; cat. no. CRL-11268) cells were maintained in Dulbecco's modified Eagle's medium (DMEM; Gibco, Gaithersburg, MD, USA) supplemented with 10% fetal bovine serum (Gibco). All cells were cultured with 5% CO_2_ at 37°C. For experiments with NAT10 inhibitors, treatment of cells with remodelin (dissolved to 100 mM with DMSO) at the necessary concentration was initiated 24 h prior to EV71 infection. Control cells were treated with the same volume of DMSO.

### Viruses

EV71 (strain XF) was obtained from the Microorganisms & Viruses Culture Collection Center, Wuhan Institute of Virology (WIV), Chinese Academy of Sciences (CAS) and amplified in Vero cells. EV71 wild-type (WT) and ac4C mutants were rescued from infectious DNA constructs in our laboratory. All viruses were titrated using 50% tissue culture infectious dose (TCID_50_) assays with the Reed–Muench formula (36).

### Mice

Six-day-old AG6 mice (37) were randomly assigned to different groups for virus infection. All animal experiments were carried out in strict accordance with institutional guidelines and were approved by the Institutional Animal Care and Use Committee of WIV, CAS (approval no. WIVA32202102).

### Plasmid constructs

**Figure 1.**
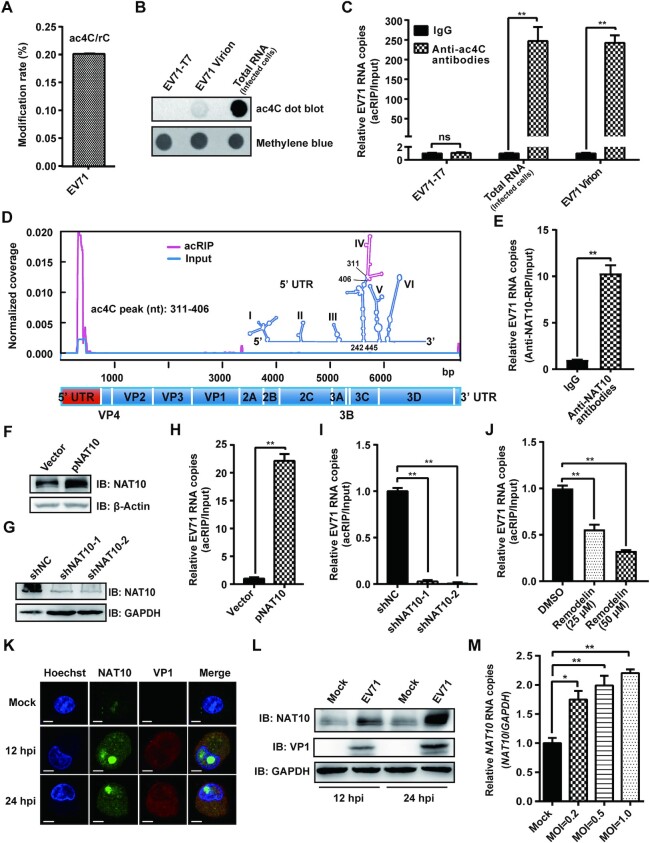
EV71 contained the ac4C modification and altered the expression pattern of NAT10. (**A**) UPLC-MS/MS of the EV71 RNA genome. EV71 RNA was extracted from viral supernatants concentrated by ultracentrifugation. The percentage of ac4C among C residues is presented on the y-axis. (**B**) Anti-ac4C dot blot of EV71 RNA from (A). Methylene blue staining was used as a loading control. The *in vitro* T7-transcribed EV71 genome and total RNA from EV71-infected Vero cells served as negative and positive controls, respectively. (**C**) acRIP-qRT-PCR. RNAs from EV71-infected cells, EV71 virions (A), and transcribed by T7 polymerase were incubated with IgG or anti-ac4C antibodies, followed by IP and qRT-PCR. Data are means ± SEMs (*n* = 3). **P ≤ 0.01, ns: not significant, unpaired Student's *t*-tests. (**D**) Mapping of ac4C sites on EV71 RNA by acRIP-Seq. Poly(A)+ RNA, purified from total RNA extracted from EV71-infected Vero cells, was fragmented, and IP was performed using anti-ac4C antibodies, followed by next-generation sequencing. Normalized coverage on acRIP-Seq and input EV71 RNA are presented in red and blue, respectively. The RNA secondary structure was predicted using Mfold software. The red line in stem–loop IV represents the peak area of ac4C. (**E**) Binding of NAT10 to EV71 RNA. EV71-infected Vero cells were crosslinked using formaldehyde, and IP was performed using anti-NAT10 antibodies. The results were quantified by qPCR. IgG was used as a negative control. Data are means ± SEMs (*n* = 3). **P ≤ 0.01, unpaired Student's *t*-tests. (**F**, **G**) Western blotting of extracts from EV71-infected Vero cells treated with pNAT10 (F) or shRNA (G). The expression of NAT10 was assessed using anti-NAT10 antibodies, and GAPDH served as a loading control. (**H**–**J**) Modulation of EV71 RNA acetylation by NAT10. Total RNA was extracted from EV71-infected Vero cells with NAT10 overexpression (H), knockdown (I), or inhibition (J); isolated using acRIP; and quantified using qRT-PCR. Data are means ± SEMs (*n* = 3). **P ≤ 0.01, unpaired Student's *t*-tests. (**K**) Confocal microscopy images of NAT10 in EV71- or mock-infected cells. The nucleus (blue), EV71 protein (red), and NAT10 (green) were labeled with Hoechst 33258, anti-VP1 antibodies, and anti-NAT10 antibodies, respectively. Scale bars, 5 μm. (**L**) Western blot analysis of extracts of EV71-infected (MOI = 1) Vero cells (12 and 24 hpi). GAPDH was used as a loading control. (**M**) RNA expression levels of *NAT10*. Total RNA was extracted at 12 hpi from EV71- (MOI = 0.2, 0.5, or 1) or mock-infected Vero cells and quantified using qRT-PCR. *GAPDH* was used as a control. Data are means ± SEMs (*n* = 3). *P ≤ 0.05, **P ≤ 0.01, unpaired Student's *t*-tests.

NAT10 eukaryotic expression plasmid (pNAT10) was constructed by inserting the coding sequence (CDS) of NAT10 from *Chlorocebus aethiops* into the vector pcDNA3.0 (Invitrogen, Carlsbad, CA, USA; Figure 1F).

**Figure 2.**
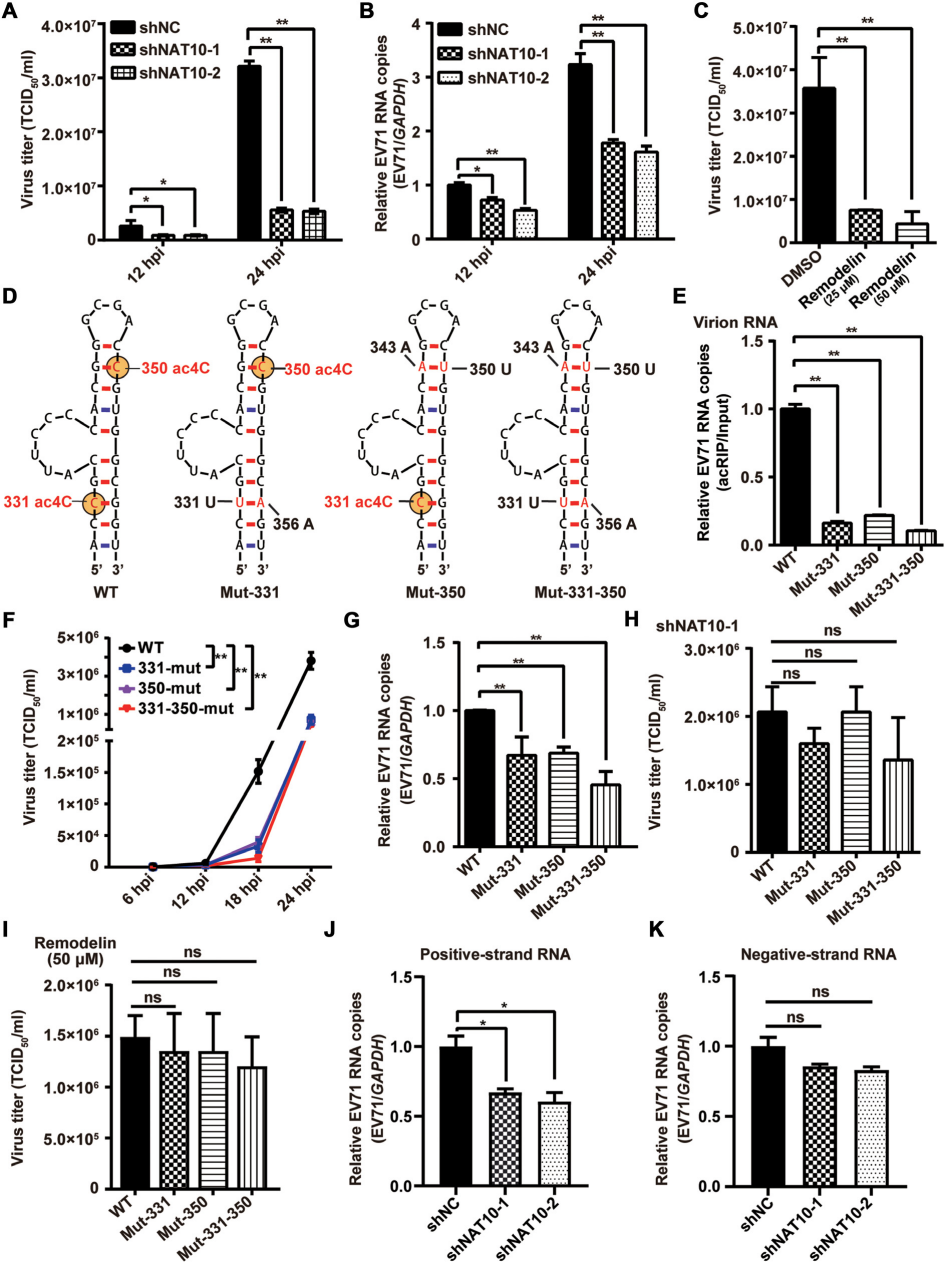
ac4C modifications promoted EV71 replication. (**A**, **C**) Viral titers (TCID_50_/ml). The supernatants of EV71-infected Vero cells with NAT10 knockdown (A) or inhibition by remodelin (C) were collected at the indicated time points postinfection, and EV71 titers were measured as the TCID_50_. Data are means ± SDs (*n* = 3). *P ≤ 0.05, **P ≤ 0.01, unpaired Student's *t*-tests. (**B**) qRT-PCR was performed to measure EV71 RNA levels in Vero cells in which NAT10 was knocked down at the indicated times postinfection. GAPDH was used as a control. Data are means ± SEMs (*n* = 3). *P ≤ 0.05, **P ≤ 0.01, unpaired Student's *t*-tests. (**D**) Schematic showing the location of ac4C sites in EV71 WT and mutants. The RNA secondary structure was predicted using Mfold software. Yellow solid circles indicate ac4C modification. (**E**) ac4C levels in EV71 WT and mutants were determined using acRIP-qRT-PCR. EV71 virion RNA was extracted from the supernatants of EV71-infected Vero cells and incubated with anti-ac4C antibodies, followed by IP and qRT-PCR. Data are means ± SEMs (*n* = 3). **P ≤ 0.01, unpaired Student's *t*-tests. (**F**) Growth curves of EV71 WT and ac4C mutants. The supernatants of EV71 WT- or ac4C mutant-infected Vero cells were harvested at the indicated times postinfection, and growth curves were plotted. Data are means ± SDs (*n* = 3). **P ≤ 0.01, two-way ANOVA. (**G**) qRT-PCR was performed to determine the RNA levels of EV71 WT or ac4C mutants in Vero cells at 24 hpi, and GAPDH was used as a control. Data are means ± SEMs (*n* = 3). **P ≤ 0.01, unpaired Student's *t*-tests. (**H**, **I**) Viral titers (TCID_50_/ml). The supernatants of EV71 WT- or ac4C mutant-infected Vero cells, treated with shNAT10 (H) or remodelin (I), were collected at 24 hpi, and EV71 titers were measured as the TCID_50_. Data are means ± SDs (*n* = 3). ns: not significant, unpaired Student's *t*-tests. (**J**, **K**) qRT-PCR was performed to determine the positive-strand (J) or negative-strand (K) RNA levels of EV71 in Vero cells treated with shNC or shNAT10 at 24 hpi, with GAPDH used as a control. Data are means ± SEMs (*n* = 3). *P ≤ 0.05, ns: not significant, unpaired Student's *t*-tests.

ac4C mutation EV71 infectious DNA constructs were generated by mutating nt 331 (pMut-331), 350 (pMut-350), both (pMut-331–350) or 383 from C to T, with the base pairing restored by G-A mutation at position 356, 343 or 390 (Figure 2D and Supplementary Figure S2F) in the infectious DNA construct (38). These nucleotides were also mutated from C to G to generate pMut-331-G, pMut-350-G, and pMut331-350-G (Supplementary Figure S2I).

**Figure 3.**
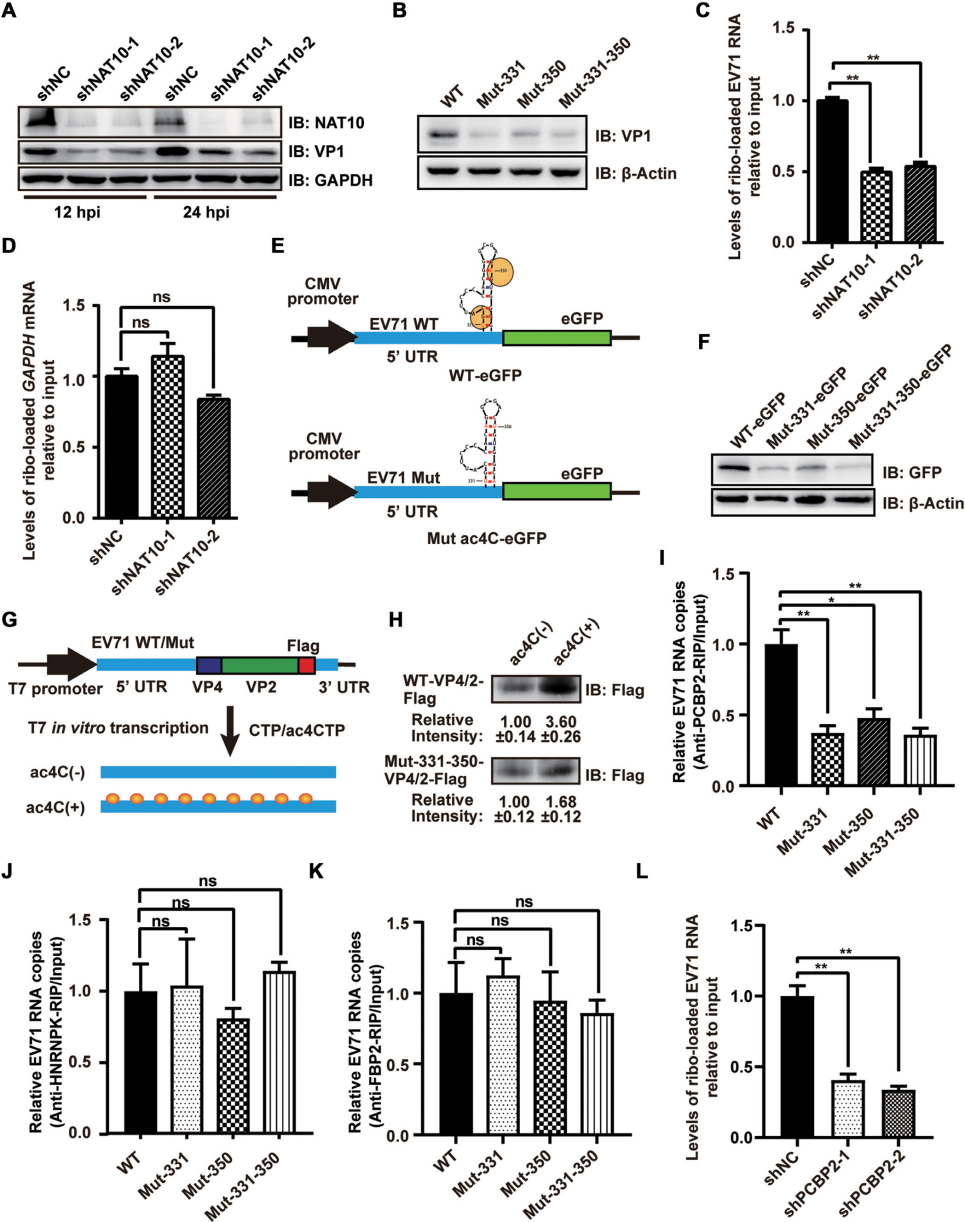
ac4C enhanced the translation efficiency of EV71 RNA. (**A**, **B**) Western blot analysis of extracts of NAT10-knockdown RD cells infected with EV71 at 12 and 24 hpi (A) or Vero cells infected with EV71 WT and ac4C mutants at 24 hpi (B). GAPDH was used as a loading control. (**C**, **D**) Vero cells with or without NAT10 knockdown were infected with EV71 and used to analyze input and ribosome-loaded RNA levels for EV71 (C) and GAPDH (D) at 24 hpi. Data are means ± SEMs (*n* = 3). **P ≤ 0.01, ns: not significant, unpaired Student's *t*-tests. (**E**) Schematic representation of the eGFP reporter vector with insertion of the 5′ UTR of EV71 WT or ac4C mutants. (**F**) Representative western blots of extracts from RD cells transfected with eGFP reporter vectors (E) at the indicated times post-transfection. β-Actin was used as a loading control. (**G**) Schematic representation of the *in vitro* translation template. Yellow solid circles indicate ac4C modification. (**H**) *In vitro* translation assays were performed using ac4C(±) RNA template. Gray intensity was quantified using ImageJ. Data are means ± SDs (*n* = 3). (**I**–**K**) Binding of PCBP2, HNRNPK, and FBP2 to RNA from EV71 WT or ac4C mutants. EV71 WT- or ac4C mutant-infected Vero cells were crosslinked with formaldehyde and subjected to IP using anti-PCBP2 (I), anti-HNRNPK (J), or anti-FBP2 (K) antibodies. Expression was quantified using qRT-PCR. Data are means ± SEMs (*n* = 3). *P ≤ 0.05, **P ≤ 0.01, ns: not significant, unpaired Student's *t*-tests. (**L**) shNC- or shPCBP2-treated Vero cells were infected with EV71 and used to analyze input RNA and ribosome-loaded RNA levels of EV71 at 24 hpi. Data are means ± SEMs (*n* = 3). **P ≤ 0.01, unpaired Student's *t*-tests.

EV71 5′ UTR-enhanced green fluorescent protein (eGFP) reporter plasmids were generated by inserting the EV71 WT or ac4C mutant 5′ UTR between the cytomegalovirus (CMV) promoter and eGFP in pEGFP-N1 (Clontech; Figure 3E).

*In vitro* translation plasmids were generated by replacing nt 1713–7322 with Flag sequence (5′-GATTACAAGGACGACGATGACAAG-3′) on EV71 WT (38) or ac4C mutant (pMut-331–350) infectious DNA constructs (Figure 3G).

**Figure 4.**
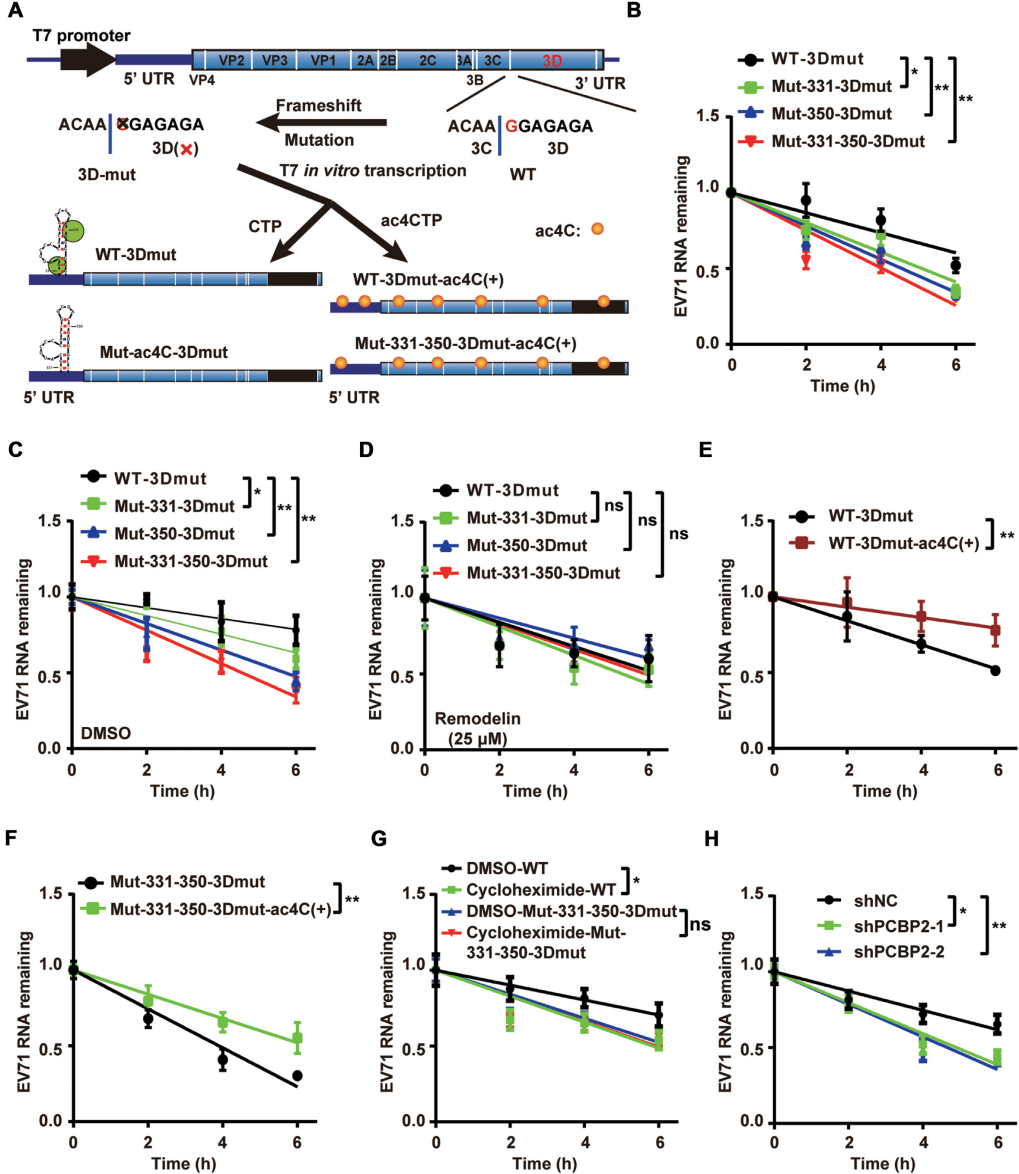
ac4C enhanced the stability of EV71 RNA. (**A**) Schematic showing that EV71 3D mutant genomes with different ac4C modification levels were produced by T7 in vitro transcription. Yellow solid circles indicate the ac4C modification. (**B**–**D**) EV71 ac4C mutants decreased the stability of EV71 RNA. T7 transcribed WT- and ac4C mutant-3Dmut viral genomes were transfected into Vero cells. qRT-PCR was performed to determine the RNA levels of WT or ac4C mutant 3D-mut viral genomes in Vero cells treated with DMSO (C), remodelin (D), or nothing (B) at the indicated times post-transfection (0 h represents 4 h after transfection). GAPDH was used as a control. Decay graphs were generated by applying the linear regression analysis. Data are means ± SEMs (*n* = 3). *P ≤ 0.05, **P ≤ 0.01, ns: not significant, two-way ANOVA. (**E**, **F**) qRT-PCR was performed to measure the RNA levels of WT-3Dmut (E) or ac4C mutant-3Dmut (F) viral genomes with (ac4C[+]) or without ac4C modification in Vero cells at the indicated times post-transfection (0 h represents 4 h after transfection). GAPDH was used as a control. Data are means ± SEMs (*n* = 3). **P ≤ 0.01, two-way ANOVA. (**G**, **H**) EV71 RNA translation efficiency affected RNA stability. qRT-PCR was performed to determine the RNA levels of WT or ac4C mutant 3D-mut viral genomes in Vero cells treated with DMSO (G), cycloheximide (G) or shRNAs (H) at the indicated times post-transfection (0 h represents 4 h after transfection). GAPDH was used as a control. Decay graphs were generated by applying the linear regression analysis. Data are means ± SEMs (*n* = 3). *P ≤ 0.05, **P ≤ 0.01, ns: not significant, two-way ANOVA.

EV71 WT- and ac4C mutant-3Dmut were constructed by deleting the first base of the 3D coding region in the corresponding infectious DNA construct (Figure 4A).

### Ultra-high-performance liquid chromatography tandem mass spectrometry (UPLC-MS/MS)

UPLC-MS/MS was performed by Wuhan Metware Biotechnology Co., Ltd. Briefly, EV71 supernatants were concentrated by ultracentrifugation in a SW28Ti rotor (Beckman Coulter) at 26 000 rpm for 2 h at 4°C. EV71 RNA was extracted using an RNeasy mini kit (Qiagen, Valencia, CA, USA) and digested into nucleosides using S1 nuclease (Takara, Shiga, Japan), alkaline phosphatase (Takara), and phosphodiesterase I (Sigma-Aldrich, St. Louis, MO, USA). The nucleosides were extracted with chloroform and analyzed using a UPLC-ESI-MS/MS system (UPLC: ExionLC AD; MS: Applied Biosystems 6500 Triple Quadrupole). RNA modifications were detected using MetWare (http://www.metware.cn/) based on the AB Sciex QTRAP 6500 LC–MS/MS platform.

### *In vitro* transcription of and transfection with EV71 RNA

EV71 genome RNA was *in vitro* transcribed from a HindIII (ThermoFisher)-linearized infectious DNA construct (38) with CTP (ThermoFisher) or ac4CTP (MedChemExpress) as substrates using a MEGAscript T7 Kit (Ambion, Austin, TX, USA) according to the manufacturer's instructions. The RNAs were transfected into Vero or RD cells with DMRIE-C Reagent (ThermoFisher) according to standard protocols.

### Dot blot

Anti-ac4C dot blot assays were performed as previously described (20). Briefly, 5 μg RNA was denatured for 5 min at 75°C, placed on ice for 1 min, loaded onto Hybond-N^+^ membranes, and subjected to ultraviolet crosslinking. ac4C detection was carried out with anti-ac4C antibodies (Abcam, Cambridge, UK) using standard protocols. Signals were detected using a ChemiDoc MP imaging system (Bio-Rad Laboratories, Berkeley, CA, USA).

### Quantitative reverse transcription polymerase chain reaction (qRT-PCR)

RNA was extracted from cells using TRIzol reagent (Invitrogen), and qRT-PCR was performed using a HiScript II One Step qRT-PCR SYBR Green Kit (Vazyme) and Hard-Shell PCR Plates (96-well; Bio-Rad Laboratories) on a CFX Connect Real-Time system (Bio-Rad Laboratories). The thermocycling parameters were as follows: 55°C for 5 min, 95°C for 30 s, and 40 cycles of 95°C for 10 s and 60°C for 45 s. For detection of EV71 positive- or negative-strand RNA, reverse transcription was first performed using HiScript 1st Strand cDNA Synthesis Kit (Vazyme) with the EV71 VP1 forward qPCR primer (for negative-strand RNA detection) or reverse primer (for positive-strand RNA detection) and qRT-PCR was then carried out using Hieff qPCR SYBR Green Master Mix (Yeasen). The thermocycling parameters were as followed: 95°C for 5 min, followed by 40 cycles of 95°C for 10 s, 55°C for 20 s, and 72°C for 20 s. Gene expression was quantified using the 2^−△△Cq^ method with Bio-Rad CFX Manager 3.0. The primers used in qRT-PCR were as follows: EV71 VP1 (forward: 5′-CGAATGCTAGTGATGAGAGTAT-3′, reverse: 5′-GAGGAAGATCTATCTCCCCAACT-3′), EV71 5′ UTR (forward: 5′-ACAATTAAAGAGTTGTTACCATATAGCTATTGGATTGGCC-3′, reverse: 5′-CATGTTTTGCTGTGTTGAGGGTCAAGAT-3′), *NAT10* (forward: 5′-GGGTATGGTGGCCCACTTAAT-3′, reverse: 5′-CCAACAAGCCTCCGTACCAT-3′), glyceraldehyde 3-phosphate dehydrogenase (*GAPDH*) for humans and monkeys (forward: 5′-GAAGGTGAAGGTCGGAGTC-3′, reverse: 5′-GAAGATGGTGATGGGATTTC-3′), and *GAPDH* for mice (forward: 5′-CATCACTGCCACCCAGAAGACTG-3′, reverse: 5′-ATGCCAGTGAGCTTCCCGTTCAG-3′). The EV71 genome was detected using the EV71 VP1 primer, and the WT- or Mut-VP4/2-Flag RNA in the *in vitro* translation system was detected using the EV71 5′ UTR primer.

### Acetylated RIP (acRIP) and acRIP-qRT-PCR

RNA was extracted from EV71-infected Vero cells and concentrated viral supernatants using TRIzol reagent (Invitrogen) or transcribed by T7 polymerase. For acRIP, 300 μg total RNA, 10 μg supernatant RNA, or 10 μg *in vitro-*transcribed EV71 RNA was incubated with anti-ac4C antibodies (Abcam) or IgG antibodies (Proteintech, Rosemont, IL, USA) in 500 μl IP buffer (150 mM NaCl, 0.1% NP-40, 10 mM Tris–HCl, pH 7.4) for 4 h at 4°C. The mixtures were incubated with 30 μl anti-rabbit antibodies conjugated with magnetic beads (NEB, Ipswich, MA, USA; cat. no. S1432S) for 2 h at 4°C and washed six times with 1 ml IP buffer. RNA was extracted using TRIzol reagent and quantified by qRT-PCR.

### acRIP-Seq

acRIP-Seq was performed by Cloudseq Biotech Inc. (Shanghai, China) according to a previously published protocol (20). Briefly, rRNA from Poly(A)+ RNA, purified from total RNA from EV71-infected Vero cells using a GenElute mRNA Miniprep Kit (Sigma-Aldrich), was removed using a Ribo-zero kit (Illumina, San Diego, CA, USA). acRIP was performed with a GenSeq ac4C RIP Kit according to the manufacturer's instructions. Both acRIP and input samples were used for library generation with a NEBNext Ultra II Directional RNA Library Prep Kit (New England Biolabs). The library was validated using an Agilent 2100 bioanalyzer and sequenced on a HiSeq 4000 instrument (Illumina). Analysis of acRIP-Seq data was consistent with MeRIP-seq, as described previously (39).

### Western blot analysis

Cells infected with EV71 or transfected with plasmids were harvested and lysed at the indicated times. The cell lysates were separated by sodium dodecyl sulfate polyacrylamide gel electrophoresis and transferred to nitrocellulose membranes. Protein detection was performed with anti-NAT10 antibodies (cat. no. 13365–1-AP; Proteintech), anti-GAPDH antibodies (cat. no. 60004-1-lg; Proteintech), anti-VP1 antibodies (40), anti-GFP antibodies (cat. no. 66002-1-Ig; Proteintech), anti-Histone H3 antibodies (cat. no. GTX122148; GeneTex), and anti-β-actin antibodies (cat. no. sc47778; Santa Cruz Biotechnology, Dallas, TX, USA) using standard protocols (41).

### Short hairpin RNA (shRNA)-mediated gene silencing

The shRNAs specific to each gene were as follows: human *NAT10* (shNAT10-1: 5′-CGGCCATCTCTCGCATCTATT-3′, shNAT10-2: 5′-GCAATTGTACACAGTGACTAT-3′), green monkey *NAT10* (shNAT10-1: 5′-CGGCCATCTCCCGCATCTATT-3′, shNAT10-2: 5′-GCAATTGTACGCAATGACCAT-3′), and green monkey monkey poly(C) binding protein 2 (*PCBP2*; shPCBP2-1: 5′-GCATTCCGCAATCCATCATTG-3′, shPCBP2-2: 5′-TCCTGAGAGAATTATCACTTT-3′). The shRNAs were cloned into pLKO.1-TRC (Addgene) using EcoRI (ThermoFisher) and AgeI (ThermoFisher) and packaged into lentiviruses using psPAX2 and pMD2.G. Stable knockdown cell lines were screened by puromycin after lentiviral infection (Vero cells: 10 μg/ml, RD cells: 2 μg/ml).

### Formaldehyde-crosslinked RIP and qRT-PCR

RIP-qRT-PCR was performed as described previously (40). Briefly, cells were transfected with pNAT10, pFlag-3D (42), or nothing and infected with EV71 or transfected with the EV71 genome. Cells were then crosslinked using phosphate-buffered saline (PBS) containing 1% formaldehyde and incubated for 10 min at 37°C. The crosslinking was terminated by 0.125 M glycine, and the cells were harvested and incubated with antibodies against NAT10 (Proteintech), Flag (Sigma-Aldrich), PCBP2 (GeneTex), far upstream element binding protein 2 (FBP2; Santa Cruz Biotechnology), heterogeneous nuclear ribonucleoprotein K (HNRNPK; Proteintech), or IgG in 500 μl RIP buffer (150 mM KCl, 25 mM Tris–HCl, pH 7.4, 5 mM ethylenediaminetetraacetic acid [EDTA], 0.5 mM dithiothreitol [DTT], 0.5% NP40, 100 U/ml RNase inhibitor, 100 μM phenylmethylsulfonyl fluoride, and 1 μg/ml proteinase inhibitor) overnight at 4°C. The following day, the mixtures were incubated with 30 μl preblocked protein-G or -A agarose beads for 2 h at 4°C and washed six times with 1 mL RIP buffer. RNA was then extracted using TRIzol reagent and quantified by qRT-PCR.

### Immunofluorescence confocal microscopy

Immunofluorescence assays were performed as described previously (43). Briefly, Vero cells were seeded to ∼50% confluence and infected with EV71. At the indicated times, cells were fixed in 4% paraformaldehyde (PFA) for 15 min, permeabilized in 0.5% Triton X-100 for 3 min, and blocked in 3% bovine serum albumin (BSA) for 1 h at room temperature. Cells were then incubated with primary antibodies overnight at 4°C, washed three times with PBS, and stained with corresponding secondary antibodies for 1 h at room temperature. Nuclei were stained with Hoechst 33258 (Invitrogen), and images were captured using a confocal microscope (Nikon).

### Quantification of ribosome-loaded EV71 RNA

Ribosome-loaded EV71 RNA was quantified as previously described (44). Briefly, NAT10-knockdown or remodelin-treated Vero cells were infected with EV71 for 12 h and then treated with 100 μg/ml cycloheximide at 37°C for 10 min. The cells were washed three times using PBS and lysed in ribosome lysis buffer (10 mM Tris–HCl, pH 7.4, 100 mM KCl, 5 mM MgCl_2_, 1% Triton X-100, 2 mM DTT, 100 μg/ml cycloheximide, 100 U/ml RNase inhibitor, and 1 μg/ml proteinase inhibitor). The lysates were sheared using a 26-gauge needle and clarified by centrifugation at 2000 × *g* for 10 min at 4°C. One-tenth of the supernatant was used as the input sample. The remaining supernatant was loaded onto a 10–45% sucrose gradient and centrifuged at 30 000 × *g* for 3 h at 4°C. RNA was extracted from the input or ribosomal pellet using TRIzol and quantified by qRT-PCR.

### *In vitro* PCBP2 binding assay

The EV71 genome was transcribed using a T7 Kit (Ambion) with CTP or ac4CTP added or extracted from the supernatant of EV71 WT- or Mut-331–350-infected Vero cells. EV71 RNA levels were quantified using qRT-PCR, and 5 μg GST-PCBP2 (Proteintech) was mixed with Glutathione High Capacity Magnetic Agarose Beads (Millipore) for 1 h at 4°C in binding buffer (20 mM Tris pH 7.5, 120 mM KCl, 1 mM DTT, 2.5 mM MgCl_2_), followed by three washes with binding buffer. Subsequently, GST-PCBP2-beads and 0.05 nmol EV71 genome were mixed in binding buffer and incubated at 37°C for 5 min, followed by six washes with binding buffer. The RNAs were then extracted with TRIzol reagent and quantified using qRT-PCR.

### *In vitro* translation assay

The RNA templates were *in vitro* transcribed from a HindIII (ThermoFisher)-linearized EV71 cDNA plasmids or from gel-purified PCR product of full-length *GAPDH* mRNA sequence with CTP (ThermoFisher) or ac4CTP (MedChemExpress) as substrates using a MEGAscript T7 Kit (Ambion). The *in vitro* translation assay was performed using a Retic Lysate IVT Kit (ThermoFisher) according to the manufacturer's instructions. The translation reaction was carried out for 1.5 h, and western blot was performed.

### *In vitro* 3D binding assay

The EV71 genome was transcribed using a T7 Kit (Ambion) with CTP or ac4CTP added or extracted from the supernatant of EV71 WT-infected Vero cells. EV71 RNA levels were quantified using qRT-PCR. RNA primers (0.5 nmol) and the EV71 genome (0.05 nmol) were mixed in RNA annealing buffer (50 mM NaCl, 5 mM Tris–HCl [pH 7.5], 5 mM MgCl_2_) in a total volume of 100 μl, denatured at 75°C for 10 min, slowly annealed, and then cooled to room temperature. The annealed solution and His-3D (45) were mixed in binding buffer (50 mM HEPES [pH 7.0], 75 mM KCl, 5 mM MgCl_2_, 4 mM TCEP) and heated at 37°C for 1.5 h. The mixture was then incubated with anti-His tag antibodies (Proteintech) overnight at 4°C, then incubated with protein-G agarose beads for 1 h and washed six times with binding buffer. RNA was extracted with TRIzol and quantified using qRT-PCR.

### Infection of mice

AG6 mice were inoculated via the intraperitoneal (i.p.) route (50 μl in DMEM; 10^5^ PFU of EV71-WT or ac4C mutant virus/mouse). Mouse morbidity was monitored by daily weighing. For histology, immunohistochemistry (IHC), and viral RNA quantification, mice were sacrificed at 2 or 3 days postinfection. All tested organs, including the heart, liver, spleen, lungs, kidneys, brain, intestines and thigh muscles, were harvested.

### Histopathological analysis and IHC

Histopathological analysis and IHC were performed by Wuhan Servicebio Technology Co., Ltd. For histopathological analysis, tissues harvested from EV71- or DMEM-challenged mice were immediately fixed in 4% PFA, embedded in paraffin, sectioned, and stained with hematoxylin and eosin. For IHC, the sections were blocked with PBS containing 3% BSA for 30 min at room temperature and then incubated with anti-VP1 antibodies (1:500) overnight at 4°C. After three washes with PBS, the sections were incubated with secondary antibodies (labeled with horseradish peroxidase) at room temperature for 50 min, and diaminobenzidine chromogenic agent was used for IHC according to standard protocols. Samples were counterstained with hematoxylin stain solution for approximately 3 min. The images were collected using Pannoramic DESK, P-MIDI and P250 (3D HISTECH).

### Quantification and statistical analysis

All data were analyzed using GraphPad Prism software (version 8). The details of the statistical tests used in this study are specified within the corresponding figure legends. Data are presented as the means ± standard error of the means (SEMs) or means ± standard deviations (SDs). Results with *P* values of 0.05 or less were considered significant.

## RESULTS

### The 5′ UTR of EV71 RNA contained ac4C modifications and the expression pattern of host acetyltransferase NAT10 was altered during infection

To investigate whether EV71 RNA contained ac4C modifications, virion RNA was purified from the infected culture at a large-scale and quantified by UPLC-MS/MS. Notably, ac4C residues in EV71 RNA accounted for 0.201% of total cytidines (Figure 1A). ac4C modifications were detected in virion RNAs and total RNAs from EV71-infected Vero cells, but not in *in vitro*-transcribed EV71 genomic RNAs, as evidenced by dot blot with an anti-ac4C antibody (Figure 1B). acRIP with anti-ac4C antibodies followed by qRT-PCR analysis using EV71 primers showed that EV71 RNAs from both virus-infected cells and virions were pulled down, whereas the viral genome transcribed *in vitro* was not (Figure 1C). These results indicated that EV71 RNA contained ac4C modifications. To map the ac4C modification status in the EV71 genome, acRIP-seq was performed. A distinct ac4C peak was identified in the region of the EV71 genome spanning from nucleotides (nt) 311 to 406 (Figure 1D), which was located in stem-loop IV (nt 242–445) within the IRES of the 5′ UTR (46). Collectively, our results demonstrated that EV71 RNA contained ac4C residues.

EV71 does not encode any protein with acetyltransferase activity. To investigate whether the ac4C modification on EV71 RNA was catalyzed by the cellular acetyltransferase NAT10, EV71-infected Vero cells were crosslinked by formaldehyde, and IP with anti-NAT10 antibodies was performed. GTF3C4, a protein with histone acetyltransferase activity and that is essential for the generation of various small RNAs by RNA polymerase III (47), was used as a control. qRT-PCR showed that EV71 RNA was pulled down by NAT10 (Figure 1E) but not by GTF3C4 (Supplementary Figure S1A), indicating an interaction of NAT10 with EV71 RNA. We subsequently examined the abundance of ac4C in EV71 RNA using acRIP-qRT-PCR in cells overexpressing NAT10 following transfection with pNAT10 (Figure 1F) or NAT10 knockdown using shRNA (Figure 1G). The intensity of ac4C on viral RNA was increased when NAT10 was overexpressed (Figure 1H) and decreased when NAT10 was knocked down (Figure 1I). In addition, inhibition of NAT10 function in Vero cells by remodelin (Supplementary Figure S1B), a drug that blocks NAT10 function at nontoxic concentrations in cell cultures and in mice (24,32,48), decreased ac4C levels in EV71 RNA (Figure 1J). Taken together, these results showed that deposition of ac4C on EV71 RNA was mediated by the host acetyltransferase NAT10.

We next investigated whether EV71 infection altered the subcellular localization of NAT10 because EV71 undergoes replication in the cytoplasm (49), whereas NAT10 is mainly distributed in the nucleus (20,50). Immunofluorescence showed that NAT10 was localized in both the nucleus and cytoplasm in the presence of EV71 (Figure 1K) and that the ratio of NAT10 in the cytoplasm to that in the nucleus was increased (Supplementary Figure S1C). Western blotting also revealed that more NAT10 was distributed in the cytoplasmic fraction after virus infection (Supplementary Figure S1D), indicating that EV71 altered the subcellular localization of NAT10. A stronger signal for NAT10 was detected during virus infection in immunofluorescence experiments (Figure 1K), implying that EV71 infection may enhance NAT10 expression. These findings were further confirmed by western blotting, in which NAT10 expression increased at 12 and 24 h postinfection (hpi; Figure 1L), accompanied by increased cellular ac4C modification (Supplementary Figure S1E). Notably, the RNA level of *NAT10* increased (Figure 1M) as the multiplicity of infection (MOI) increased (Supplementary Figure S1F).

### ac4C modifications in the 5′ UTR promoted EV71 replication

To investigate whether ac4C affected EV71 replication, NAT10 in Vero cells was knocked down (Figure 1G) using shRNA or inhibited by remodelin, followed by EV71 infection. Interestingly, both virus titers (Figure 2A) and EV71 RNA copy numbers (Figure 2B) were significantly decreased at 12 and 24 hpi when NAT10 was silenced (Figure 2A and B). Moreover, EV71 titers (Figure 2C) and RNA levels (Supplementary Figure S2A) were decreased as the remodelin concentration increased (Figure 2C and Supplementary Figure S2A). Consistent with these findings, remodelin attenuated the viral-induced cytopathy effect (Supplementary Figure S2B). Taken together, these results suggested that NAT10 promoted virus replication.

NAT10-type acetyltransferases act at the consensus motif 5′-CCG-3′, promoting acetylation of the central cytidine (14,21–23). We then assessed whether the two 5′-CCG-3′ motifs in the ac4C peak (Figures 1D and 2D) on the EV71 5′ UTR were ac4C modified. C-T mutations were generated in the full-length EV71 cDNA clone at positions 331 (Mut-331), 350 (Mut-350), or both (Mut-331–350) with the stem-loop structure restored by G-A mutations at positions 356, 343 or both (Figure 2D). nt383 in stem-loop IV was mutated as a control (Mut-383, Supplementary Figure S2F). WT and mutant viruses were rescued, and the RNAs were subjected to acRIP-qRT-PCR. The results showed that the ac4C abundance in EV71 RNA for both ac4C mutant virions and mutant virus-infected cells was significantly decreased compared with that of WT EV71 (Figure 2E and Supplementary Figure S2C). These results indicated that these C residues were acetylated in the EV71 genome.

We next explored whether the ac4C modifications affected virus replication. To this end, Vero cells were infected by WT and ac4C mutant viruses. Consistent with the results in NAT10 knockdown/inhibition experiments (Figure 2A–C, Supplementary Figure S2A and B), both progeny virus titers (Figure 2F and Supplementary Figure S2D) and genomic RNA copies (Figure 2G) were significantly decreased when ac4C sites were mutated, whereas mutation at nt383 did not affect the viral titer and RNA copies (Supplementary Figure S2G and S2H). However, no difference in progeny virus titers was observed among WT and mutant viruses in cells with shRNA-mediated NAT10 knockdown (Figure 2H and Supplementary Figure S2E) or in the presence of remodelin (Figure 2I). Furthermore, C-G mutations at positions 331, 350, or both (Supplementary Figure S2I) also resulted in reduced ac4C levels (Supplementary Figure S2J) and replication (Supplementary Figure S2K) of EV71. These results supported that ac4C played an important role in EV71 replication.

Owing to the presence of positive- and negative-strand RNA during EV71 replication, we then examined the effects of NAT10 on different viral RNAs. Knockdown of NAT10 reduced the level of viral positive-strand RNA more significantly than that of negative-strand RNA (Figure 2J and K). Similar results were detected when cells were treated with remodelin (Supplementary Figure S2L and S2M), indicating that NAT10 mainly affected the viral positive-strand RNA.

### ac4C modification facilitated RNA translation by enhancing the binding of PCBP2 to the IRES

The acetylation sites in EV71 RNA are located in stem-loop IV (nt 242–445) within the IRES, which is crucial for viral protein synthesis (46); therefore, ac4C modification may affect the translation efficiency of EV71 RNA. VP1 protein expression was significantly reduced when NAT10 was knocked down (Figure 3A) or in ac4C mutant viruses (Figure 3B). The results indicated that the ac4C modification was linked to the expression of VP1. To further determine whether the ac4C modifications affected translation efficiency, we measured the loading efficiency of ribosomes on viral RNAs in the presence or absence of NAT10 with sucrose density gradient centrifugation followed by qRT-PCR. Notably, shRNA-mediated knockdown of NAT10 or remodelin-dependent inhibition of NAT10 significantly reduced ribosome loading onto EV71 RNA (Figure 3C and Supplementary Figure S3A), whereas ribosome binding to *GAPDH* mRNA (Figure 3D and Supplementary Figure S3B), which lacks ac4C sites (20), was not affected. Moreover, mutation at nt383 did not affect ribosome loading onto EV71 RNA or the expression of VP1 (Supplementary Figure S3C and S3D). These results indicated that ac4C enhanced the translation of EV71 RNA.

To further confirm whether ac4C was linked to RNA translation, we constructed a reporter plasmid by insertion of the 5′ UTR of EV71 WT or ac4C mutants upstream of the eGFP expression cassette (Figure 3E) and transfected these plasmids into RD cells. We found that eGFP expression was reduced in the 5′ UTR of ac4C mutants compared with that of the WT virus (Figure 3F, Supplementary Figure S3F, and S3G). These results suggested that ac4C enhanced the translation efficiency of EV71 RNA. To further confirm these results, RNA templates containing WT or ac4C mutant 5′ UTR, VP4/2, Flag and 3′ UTR were prepared by *in vitro* transcription with CTP or ac4CTP (Figure 3G) followed by *in vitro* translation. The *GAPDH* mRNA was *in vitro* transcribed and served as a control. More VP4/2-Flag expression was detected in the presence of ac4CTP in both WT and mutant 5′ UTR RNA templates (Figure 3H). Similarly, the presence of ac4C enhanced *GAPDH* expression (Supplementary Figure S3E). These data implied that ac4C promoted the translation of EV71 RNA *in vitro*. Furthermore, the presence of ac4C enhanced translation in the WT more obviously than in the mutant (Figure 3H), indicating that site-specific ac4C modification had an important function in translation.

We next studied the detailed mechanisms through which ac4C affected protein translation. PCBP2 and HNRNPK are ITAFs that can bind to stem-loop IV. PCBP2 promotes the translation of viral RNA (51,52), whereas HNRNPK is not essential for viral protein translation (46). To check whether ac4C modifications facilitated the binding of ITAFs to the IRES, PCBP2- and HNRNPK-bound RNAs were pulled down from ac4C mutant virus- or WT virus-infected cells using specific antibodies. Notably, PCBP2 pulled down more WT EV71 RNA than mutant virus RNA (Figure 3I), supporting that ac4C promoted the binding of PCBP2 to the EV71 IRES. However, the binding of HNRNPK was not affected by ac4C mutation (Figure 3J). In addition, ac4C modification did not affect the binding of EV71 RNA to FBP2 (Figure 3K), another ITAF for EV71 that cannot bind to stem-loop IV and negatively regulates viral translation (53). To further confirm the function of ac4C in the interaction between PCBP2 and the IRES, GST-PCBP2 and EV71 genomes were incubated *in vitro*. RIP and qRT-PCR showed that the EV71 genome transcribed by T7 polymerase with ac4CTP as a substrate had stronger binding ability to GST-PCBP2 compared with that of transcribed with CTP (Supplementary Figure S3H). The binding of GST-PCBP2 to WT viral particle RNA was stronger than that to ac4C mutant viral RNA (Supplementary Figure S3H), suggesting that ac4C enhanced the binding of PCBP2 to the EV71 IRES *in vitro*. Knockdown of PCBP2 (Supplementary Figure S3I) reduced the translation efficiency of EV71 RNA (Figure 3L and Supplementary Figure S3J). These results demonstrated that ac4C facilitated viral translation by selectively enhancing the binding of PCBP2 to the IRES.

### The ac4C modification boosted the stability of EV71 RNA

The translation and decay of mRNA are intricately linked. Decreased mRNA translation reduces the stability of mRNA, which reciprocally decreases translation (20,54). Therefore, we next explored whether ac4C modification was linked to EV71 RNA stability. To this end, we constructed replication-deficient plasmids of EV71 WT and ac4C mutants with a 3D frameshift mutation (Figure 4A). All plasmids were *in vitro* transcribed, and the 3Dmut EV71 RNAs were transfected into Vero cells. qRT-PCR analysis was performed at different time points. The results showed that the RNA decay of ac4C mutants was accelerated compared with that of WT virus (Figure 4B and C). However, there were no significant differences in the RNA degradation rates between WT and mutant viruses when the function of NAT10 was inhibited using remodelin (Figure 4D). The above results indicated that ac4C modification affected the RNA decay efficiency. To further confirm these results, WT-3Dmut and Mut-331–350-3Dmut plasmids were *in vitro* transcribed with CTP or ac4CTP as the substrate, and RNA decay was measured. Notably, the RNA stability of both WT-mut3D and Mut-331–350-mut3D was increased in the presence of the ac4C modification (Figure 4E and F). These data demonstrated that ac4C enhanced the stability of EV71 RNA.

We next explored the effects of viral RNA translation on RNA stability. When cycloheximide was used to treat cells to inhibit translation, the degradation of WT-3Dmut RNA was accelerated, whereas the degradation rate of ac4C mutant RNA did not change significantly (Figure 4G). The ac4C mutant viral RNAs degraded faster than WT RNA in the presence or absence of cycloheximide (Figure 4G). Notably, knockdown PCBP2 resulted in accelerated degradation of viral RNA (Figure 4H). Furthermore, we detected the levels of RNAs in the *in vitro* translation system (Figure 3G and H) and found that ac4C enhanced the stability of RNA in *in vitro* (Supplementary Figure S4A and S4B). These results suggested that the effects of ac4C on the stability of EV71 RNA were linked to the translation efficiency of viral RNA.

### ac4C promoted the binding of EV71 RNA to 3D *in vivo* and *in vitro*

**Figure 5.**
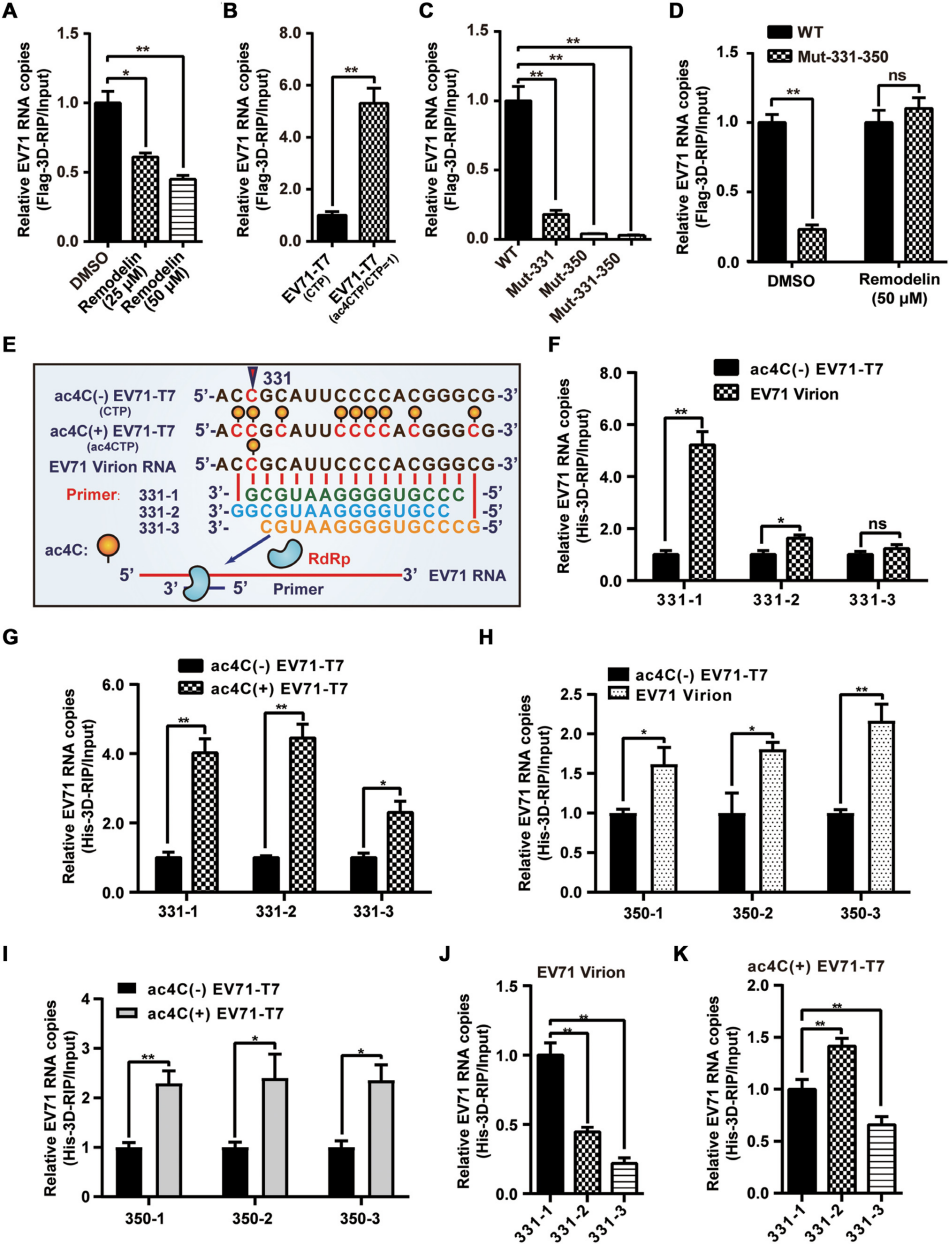
ac4C promoted the binding of EV71 RNA to 3D. (**A**, **D**) Binding of 3D to EV71 RNA following NAT10 inhibition using remodelin. Vero cells overexpressing Flag-3D were treated with DMSO or remodelin and infected with EV71 (A) or EV71 WT and ac4C mutants (D). Crosslinking with formaldehyde and IP using anti-Flag antibodies were performed, followed by quantification using qPCR. Data are means ± SEMs (*n* = 3). *P ≤ 0.05, **P ≤ 0.01, ns: not significant, unpaired Student's *t*-test. (**B**, **C**) Binding of 3D to EV71 RNA with different ac4C modification levels. Vero cells with Flag-3D overexpression were transfected with EV71 genomes with (ac4CTP/CTP = 1) or without (CTP) ac4C (B) or infected with EV71 WT and ac4C mutants (C). The same treatment as used in (A) and (D). Data are means ± SEMs (*n* = 3). *P ≤ 0.05, **P ≤ 0.01, unpaired Student's *t*-test. (**E**) Schematic of *in vitro* RNA binding assays near position 331. Yellow solid circles indicate the ac4C modification. (**F**–**K**) Binding of 3D and 331 or 350 primer-EV71 RNA pairs *in vitro*. EV71 RNAs extracted from T7 transcripts (ac4C[±]) or virions were annealed with 331- or 350–1/2/3 primers. His-3D was added, and samples were subjected to IP with anti-His antibodies, followed by quantification using qRT-PCR. Data are means ± SEMs (*n* = 3). *P ≤ 0.05, **P ≤ 0.01, ns: not significant, unpaired Student's *t*-tests. Data represent the comparison of 3D binding levels between ac4C(-) EV71-T7 and virion RNA (F & H) or ac4C(+) EV71-T7 (G & I). Alternatively, data show the binding levels of different 331 primers annealed with virion RNA (J) and ac4C(+) EV71-T7 (K).

3D is a key protein that directly binds to and synthesizes viral RNA during the replication of EV71. As acetylation promoted the binding of PCBP2 to EV71 RNA, we next evaluated whether ac4C affected the binding of 3D to viral RNA. Vero cells were transfected with the Flag-3D plasmid, subjected to EV71 infection, and cultured with 25 or 50 μM remodelin. RIP was performed with anti-Flag antibodies, and RNA binding to 3D was analyzed by qRT-PCR. We found that 3D-bound EV71 RNA was reduced by remodelin treatment (Figure 5A), implying that ac4C modification affected the binding of viral RNA to 3D. The same phenomenon was observed in both Vero and RD cells transfected with T7-transcribed EV71 RNA without ac4C (Figure 5B, Supplementary Figure S5A, and S5B) or infected with ac4C mutant viruses (Figure 5C and Supplementary Figure S5C). However, 3D-bound RNA was not significantly changed when ac4C mutant viruses were cultured with remodelin (Figure 5D). These data demonstrated that ac4C modification promoted the binding of EV71 RNA to 3D.

EV71 RNA synthesis normally starts with a tyrosine residue on the VPg (3B) protein, but is able to initiate on RNA primer-template pairs using purified 3D *in vitro* (55). To explore whether the ac4C modification affected 3D binding to the viral genome *in vitro*, RNAs were prepared by either extraction from EV71 virions or by T7 *in vitro* transcription with EV71 infectious DNA constructs with CTP or ac4CTP as the substrate (ac4C[-]-EV71-T7, ac4C[+]-EV71-T7, Figure 5E). RNA primers specific for C331 and C350 ac4C sites were designed for 3D *in vitro* binding tests (Figure 5E and Supplementary Figure S5D). 3D can bind to the double-stranded region formed by the primer annealing to the EV71 genome (Figure 5E). Interestingly, EV71 virion RNA (Figure 5F and H) and ac4C(+)-EV71-T7 (Figure 5G and I) showed stronger binding to 3D compared with ac4C(-)-EV71-T7. Compared with ac4C(-) transcripts, virion RNA and ac4C-modified *in vitro* transcripts showed different binding efficiency to 3D when paired with the primers at nt 331 (Figure 5J, K, and Supplementary Figure S5E), which was distinct from that of primers at nt 350 (Supplementary Figure S5F). These results not only suggested that ac4C enhanced 3D binding *in vitro* but also implied that the location of the acetylation site was important for the binding capacity.

### EV71 ac4C mutant virus displayed reduced pathogenicity in mice

**Figure 6.**
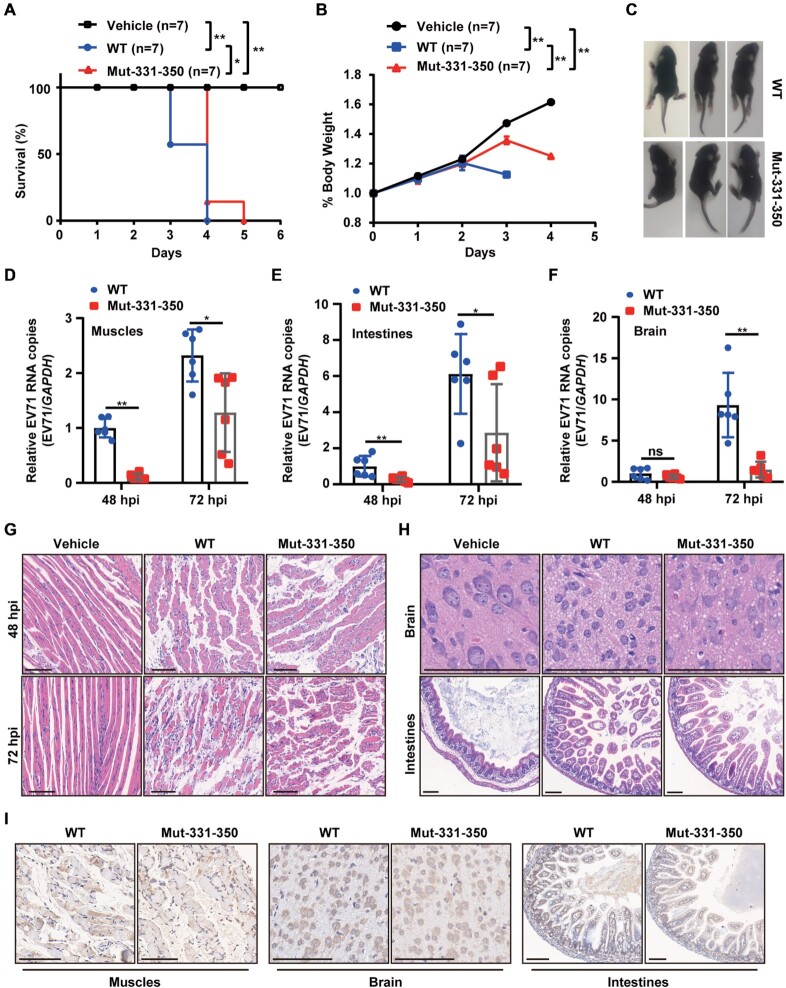
ac4C mutant virus exhibited reduced pathogenicity in mice. (**A**) Survival of mice challenged with EV71 WT or ac4C mutant (i.p., 10^5^ PFU). Control mice received DMEM. Seven mice were used per group. *P ≤ 0.05, **P ≤ 0.01, Log-rank (Mantel-Cox) tests. (**B**) Daily weights of mice from (A). **P ≤ 0.01, two-way ANOVA. (**C**) Symptoms of mice challenged with EV71 WT or ac4C mutant on day 3 postinfection (i.p., 10^5^ PFU). Symptoms included reduced motility and hind limb paralysis. (**D**–**F**) Viral RNA in the limb muscles (D), intestines (E), and brain (F) of AG6 mice infected with EV71, as quantified by qRT-PCR. Data are means ± SEMs (*n* = 6). *P ≤ 0.05, **P ≤ 0.01, ns: not significant, unpaired Student's *t*-tests. (**G**, **H**) Hematoxylin and eosin staining of the limb muscles (48 and 72 hpi) (G), brain, and intestines (72 hpi) (H). Scale bars, 100 μm. (**I**) Detection of EV71 particles in the limb muscles, intestines, and brain (72 hpi) using immunohistochemistry. Scale bars, 100 μm.

Methylation modification of IAV has been shown to be linked with disease (56). Therefore, we examined whether acetylation affected the pathogenicity of the virus. AG6 interferon receptor-deficient mice were infected with EV71 WT or Mut-331–350. Mice infected with ac4C mutant virus showed longer survival times (Figure 6A) and slower weight loss (Figure 6B) than those infected with WT virus. More severe limb paralysis was observed in mice infected with WT virus (Figure 6C). Furthermore, qRT-PCR analysis showed that the viral RNA level was significantly reduced in all tested organs, including the muscles, intestines, brain, heart, liver, spleen, lungs, and kidneys, of mice infected with Mut-331–350 virus (Figure 6D–F and Supplementary Figure S6A–E). Reduced pathogenicity was observed in the limb muscles, brain, and intestines of Mut-331–350-infected mice (Figure 6G and H), resulting in decreased muscle fiber necrosis (Figure 6G) and neuronal injury (Figure 6H), as demonstrated by hematoxylin and eosin staining. Hyperplasia, cytolysis of intestinal villi epithelial, and vacuolization of villi apical cells were decreased in intestinal cells (Figure 6H). In addition, less VP1 was detected in the muscles, intestines, and brain of Mut-331–350-infected mice by IHC when using anti-VP1 antibodies (Figure 6I). Collectively, these results demonstrated that the EV71 ac4C mutation reduced pathological damage in mice.

## DISCUSSION

Methylation modifications, such as m6A, m5C, and Nm, have been extensively studied in retroviruses, simian virus 40, Kaposi's sarcoma-associated herpesvirus, influenza virus, flavivirus, EV71, and severe acute respiratory syndrome coronavirus 2 (SARS-CoV-2) (39,40,56–66) and have been shown to play important roles in viral infection. However, little is known about the function of acetylation. In the current study, we uncovered a critical role for ac4C and its cellular machinery in regulating the replication of cytoplasmic virus EV71. ac4C in the 5′ UTR of the EV71 genome regulated RNA translation, stability, and binding ability to 3D. In addition, ac4C mutant viruses displayed slower growth and reduced pathogenicity *in vivo*.

The distribution of ac4C in EV71 was different from that in mRNA (20) or in HIV-1. ac4C was enriched in both the 5′ UTR and the translation start sites within the CDS in mRNA. In HIV-1, ac4C mainly existed in the CDS region and the 3′ UTR, not in the 5′ UTR (32). Similar to rRNA and tRNA (21–23), ac4C in EV71 was located in the stem-loop structure. Mutation the single ac4C site (nt 331 or nt 350) or both of these sites (Mut-331-G, Mut-350-G, and Mut-331–350-G) altered the secondary structure of the stem-loop, leading to stronger effects on reduced virus replication than observed in ac4C mutants that did not destroy the secondary structure (Mut-331, Mut-350, and Mut-331–350). These results implied that both the ac4C modifications and secondary structure played important roles in virus replication.

EV71 acetylation was catalyzed by host NAT10, which in turn promoted viral infection. NAT10-mediated acetylation of tRNA requires the help of the adaptor protein THUMPD1, which harbors an RNA binding motif (18,23). Whether THUMPD1 is involved in the formation of EV71 acetylation need to be further studied. Similar to METTL3 (40), the expression and localization of NAT10 were altered after EV71 infection, implying that viral infection may affect the host modification machinery to facilitate its replication. Indeed, alteration of the NAT10 expression pattern is also related to gene damage, carcinogenesis, and temperature stimulation (23,67,68), indicating that NAT10 and ac4C mediate cellular responses to various stresses.

Knockdown or inhibition of NAT10 and mutation of the ac4C site resulted in a moderate decrease in the titer of the progeny virus, suggesting that ac4C promoted virus replication. ac4C modification is one of the key factors to regulate virus replication. In the absence of ac4C, other compensatory pathways may also be activated to facilitate virus replication.

ac4C in mRNA promotes translation and stability (20), whereas its presence in HIV-1 RNA promotes viral RNA stability but does not affect viral translation (32). Our results demonstrated that ac4C in the EV71 5′ UTR promoted both the translation efficiency and the stability of viral RNA. The mechanisms through which ac4C modulated EV71 translation were different from those of mRNA. In the mRNA 5′ UTR, ac4C enhances upstream initiation and inhibits canonical start codons (69). However, ac4C in the 5′ UTR of EV71 enhanced viral protein translation efficiency by promoting the binding of the EV71 IRES to PCBP2 but did not affect binding of the IRES to FBP2 or HNRNPK. Cycloheximide treatment followed by qRT-PCR showed that WT virus RNA degraded faster, whereas the stability of mutant viral RNA was not affected, indicating that the reduced RNA stability may be a consequence of the decreased translation. Notably, Mut-331–350-3Dmut-ac4C(+) RNA is more stable than Mut-331–350-3Dmut, indicating that in addition to sites 331 and 350, the occurrence of ac4C at other sites also boosted RNA stability.

Chemical modification is closely linked to the function of virus RdRp. RdRp plays key roles in virus replication, and methyltransferases interact with RdRp to regulate its function via various mechanisms (40,66). METTL3 interacts with 3D and regulates the expression and function of 3D by ubiquitination and sumolyation (40). Moreover, the RdRp of SARS-CoV-2 interacts with METTL3 and regulates the modification of METTL3 via an unknown mechanism (66). NAT10 did not interact with 3D (data not shown). ac4C promoted the binding of 3D to EV71 RNA both *in vivo* and *in vitro*. When ac4C was paired with the last base at the 3′ end of the 331-primer *in vitro*, the affinity of 3D to viral RNA was obviously enhanced. Notably, ac4C promoted viral translation when 3D was mutated by frameshift (Supplementary Figure S7), indicating that RNA synthesis and protein translation regulated by ac4C may be two independent processes.

IAV HA m6A mutants have been reported to show reduced pathogenicity. The survival time of m6A mutant virus-infected mice was longer and weight loss was slower than in WT-infected mice (56). We also observed longer survival times and slower weight loss in ac4C mutant EV71-infected mice. Furthermore, the ac4C mutation attenuated EV71 replication *in vivo* and alleviated organ injury. These results indicated that ac4C modulated the pathogenicity of EV71. ac4C mutants decreased the replication of EV71, which could be the reason for the reduced multi-organ pathogenicity in mice. Considering the effects of m6A mutations on IAV, these results highlighted that chemical modification may be critical for viral pathogenicity. Further studies are required to evaluate the effects of the ac4C mutant virus on normal mice because AG6 mice are immunodeficient (37).

In conclusion, our results showed that ac4C played critical roles in viral infection and demonstrated that EV71 interacted with the host ac4C modification system to modulate replication. ac4C enhanced EV71 replication by boosting RNA translation, RNA stability and RNA binding to 3D. Moreover, the ac4C mutant showed reduced pathogenicity in mice. However, additional work is required to elucidate the molecular mechanisms through which ac4C regulates viral RNA function, e.g. identification of the factors involved in regulating viral RNA stability and evaluation of the effects of ac4C on conformational changes in 3D. Our data revealed the essential functions of ac4C in RNA virus replication in the cytoplasm and provided a promising target for the prevention and treatment of EV71 infection.

## DATA AVAILABILITY

The complete CDS sequence of EV71 (strain XF) used in this work has been deposited with National Center for Biotechnology Information under accession number JQ804832.

## Supplementary Material

gkac675_Supplemental_File
