# Supplementary material for: N4-acetylcytidine regulates the replication and pathogenicity of enterovirus 71
Source: Nucleic Acids Res. 2022 Aug 16;50(16):9339–54. doi: 10.1093/nar/gkac675 (PMC9458434; doi:10.1093/nar/gkac675)
Supplement: gkac675_Supplemental_File [file gkac675_supplemental_file.pdf]

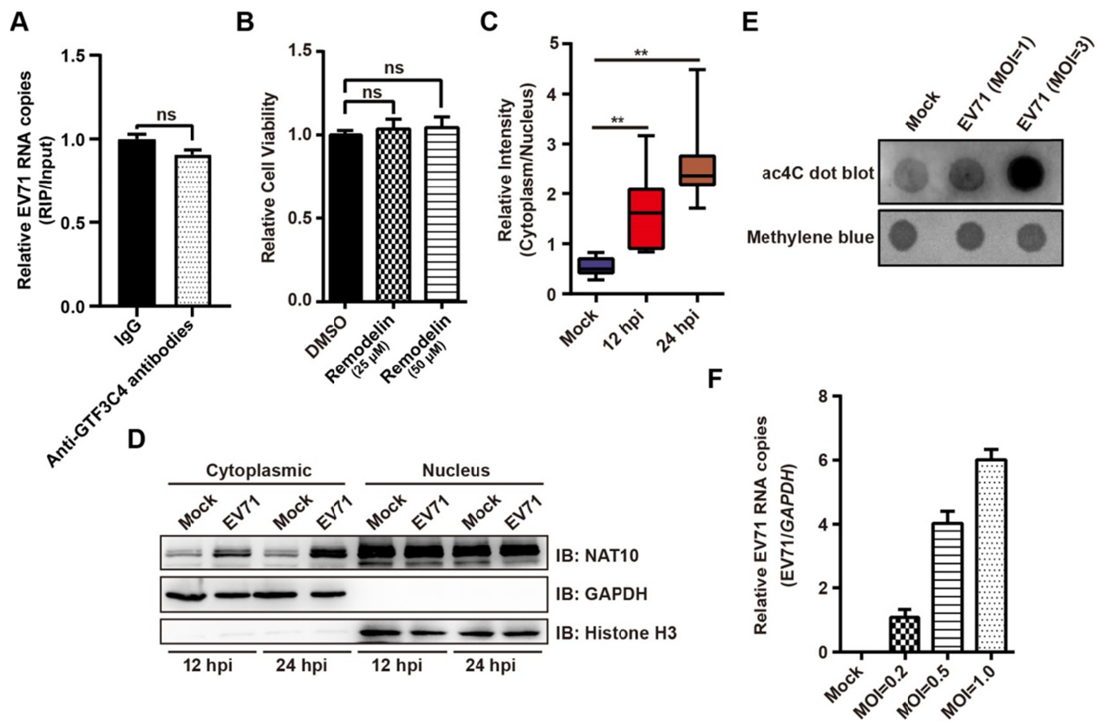

Figure S1. EV71 altered the expression pattern of NAT10. (**A**) Binding of GTF3C4 to EV71 RNA. EV71-infected Vero cells were crosslinked using formaldehyde, and IP was performed using anti-GTF3C4 antibodies. The results were quantified using qPCR. IgG was used as a negative control. Data are means ± SEMs (n = 3). ns, not significant, unpaired Student's t-tests. (**B**) DMSO- or remodelin-treated Vero cells were cultured for 1 day and their viability measured by CCK-8 Cell Counting Kit. Data are means ± SEMs (n = 3). ns, not significant, unpaired Student's t-tests. (**C**) Ratio of NAT10 in the cytoplasm to that in the nucleus, as quantified using ImageJ. Data were graphed in box-and-whisker plots, indicating the minimum, first quartile, median, third quartile, and maximum ($n \geq 10$). $^{**}P \leq 0.01$, unpaired Student's t-tests. (**D**) Localization of NAT10 in the nucleus and cytoplasm of mock- or EV71-infected Vero cells. Nuclear and cytoplasmic fractions were subjected to western blotting using antibodies against NAT10. Histone 3 and GAPDH were used as controls for each fraction. (**E**) Anti-ac4C dot blots for total RNA extracted from EV71-infected cells (MOI = 1 or 3) or mock-infected cells at 12 hpi. Methylene blue staining was used as a loading control. (**F**) RNA expression levels of EV71. Total RNA was extracted at 12 hpi from EV71- (MOI = 0.2, 0.5, or 1) or mock-infected Vero cells and quantified using qRT-PCR. *GAPDH* was used as a control. Data are means ± SEMs (n = 3).

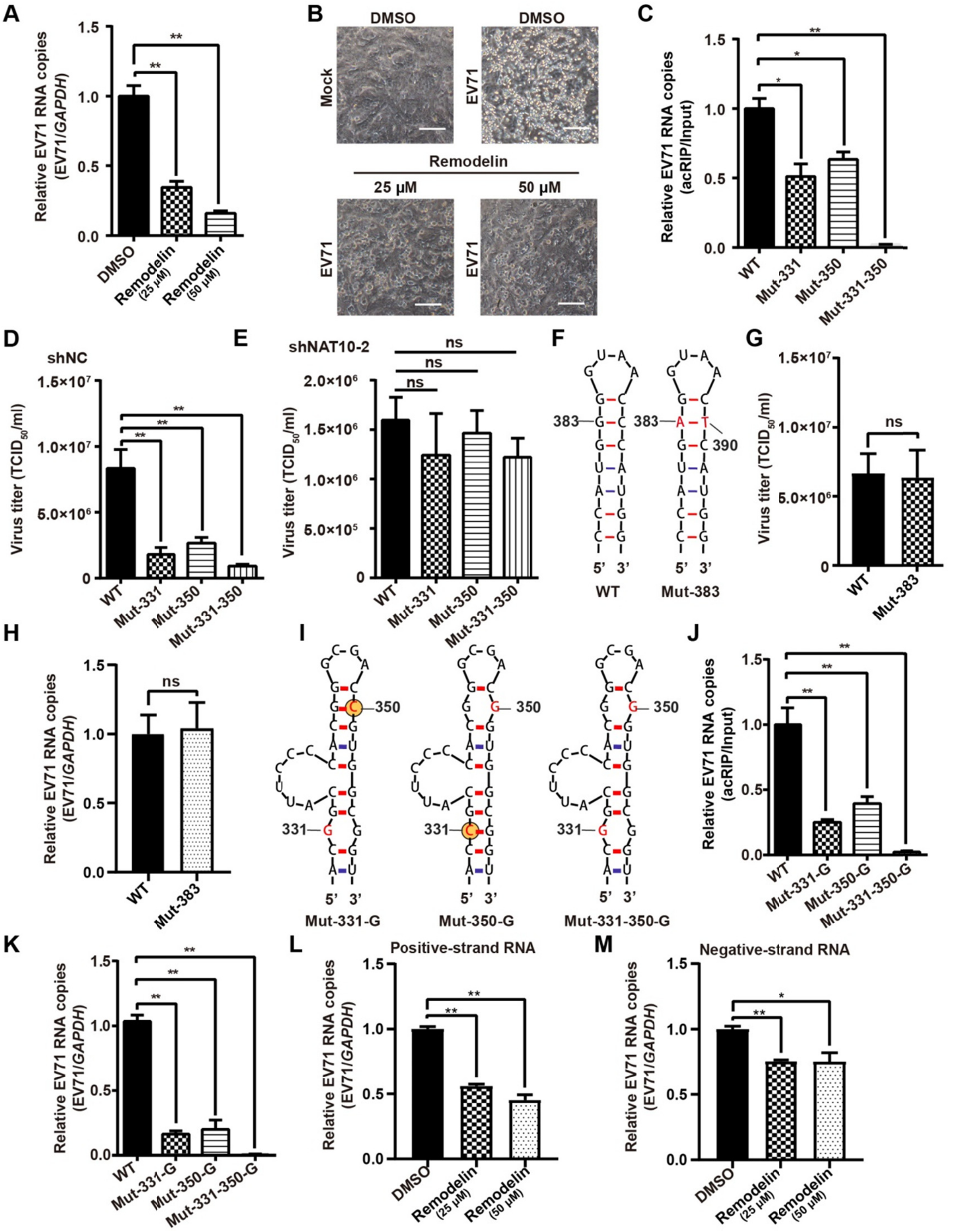

Figure S2. ac4C modification regulated EV71 replication. (**A**) qRT-PCR was performed to measure EV71 RNA levels in Vero cells in which NAT10 was inhibited by remodelin at the indicated times post-infection. *GAPDH* was used as a control. Data are means ± SEMs (n = 3). $^{**}P \leq 0.01$, unpaired Student's *t*-tests. (**B**) CPE of mock- or EV71-infected Vero cells with DMSO or remodelin treated at 12 h post-infection. Scale bars, 50 µm. (**C**) acRIP-qRT-PCR was performed to check ac4C levels of WT and mutation virus. Total RNAs were extracted from the EV71 WT- or mutants-infected Vero cells and incubated with ac4C-specific antibody, followed by IP and qRT-PCR. Data are means ± SEMs (n = 3). $^{*}P \leq 0.05$, $^{**}P \leq 0.01$, unpaired Student's *t*-tests. (**D** & **E**) Viral titers ($TCID_{50}$/mL). The supernatants of EV71 WT- or ac4C

mutant-infected Vero cells, treated with shNC (D) or shNAT10 (E), were collected at 24 hpi, and EV71 titers were measured as the $TCID_{50}$. Data are means ± SDs (*n* = 3). $^{**}P \leq 0.01$, ns: not significant, unpaired Student's *t*-tests. (**F**) Schematics of EV71 WT and Mut-383. The RNA secondary structure was predicted by Mfold software. (**G**) Viral titers ($TCID_{50}$/mL). The supernatants of EV71 WT- and Mut-383-infected Vero cells were collected at 24 hpi, and EV71 titers were measured as the $TCID_{50}$. Data are means ± SDs (*n* = 3). ns: not significant, unpaired Student's *t*-tests. (**H**) qRT-PCR was performed to determine the RNA levels of EV71 WT or Mut-383 in Vero cells at 24 h post-transfection, with *GAPDH* used as a control. Data are means ± SEMs (*n* = 3). ns: not significant, unpaired Student's *t*-tests. (**I**) Schematics of the location of ac4C sites in EV71 WT and C-G mutations. The RNA secondary structure was predicted by Mfold software. Yellow solid circles indicate ac4C modification. (**J**) ac4C levels of EV71 WT and mutations in EV71 RNA-transfected cells were determined by acRIP-qRT-PCR. Data are means ± SEMs (*n* = 3). $^{**}P \leq 0.01$, unpaired Student's *t*-tests. (**K**) qRT-PCR was performed to determine the RNA levels of EV71 WT or ac4C mutants in Vero cells at 24 h post-transfection, with *GAPDH* used as a control. Data are means ± SEMs (*n* = 3). $^{**}P \leq 0.01$, unpaired Student's *t*-tests. (**L** & **M**) qRT-PCR was performed to determine the positive-strand (L) and negative-strand (M) RNA levels of EV71 in Vero cells, treated with DMSO or remodelin at 24 hpi, with *GAPDH* used as a control. Data are means ± SEMs (*n* = 3). $^{*}P \leq 0.05$, $^{**}P \leq 0.01$, unpaired Student's *t*-tests.

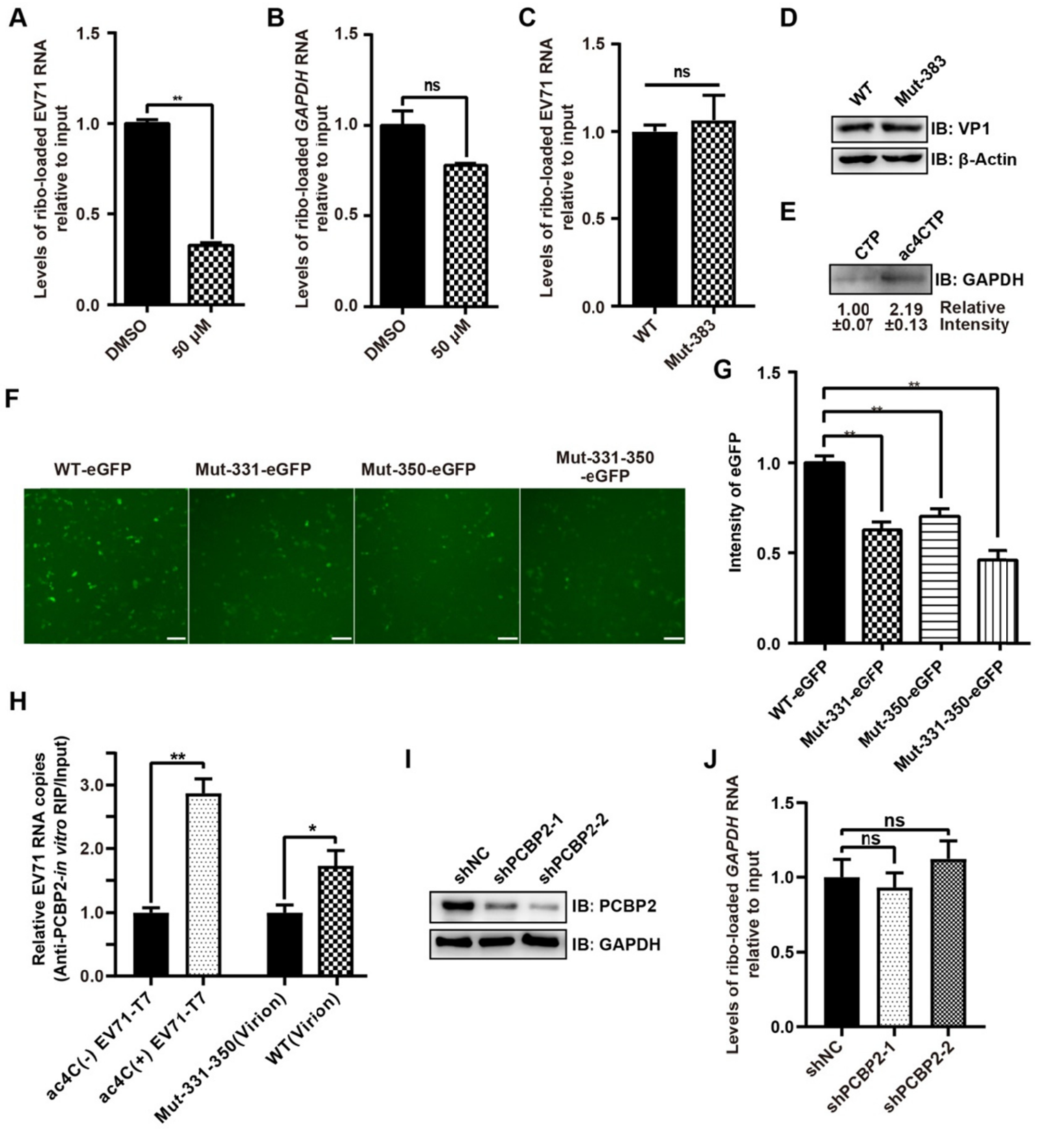

Figure S3. ac4C promoted the translation of EV71 RNA. (**A** & **B**) DMSO- or remodelin-treated Vero cells were infected by EV71 and used to analyze both the input and ribosome loaded RNA levels of EV71 (A) and GAPDH (B) at 24 h postinfection. Data are means ± SEMs (n = 3). **$P \leq 0.01$, ns, not significant, unpaired Student's *t*-tests. (**C**) EV71 WT- or Mut-383-infected Vero cells were used to analyze the input RNA and ribosome-loaded RNA levels of EV71 at 24 h postinfection. Data are means ± SEMs (n = 3). ns, not significant, unpaired Student's *t*-tests. (**D**) Western blot analysis of extracts of EV71 WT- or Mut383-infected Vero cells. GAPDH was used as a loading control. (**E**) *In vitro* translation assays were performed using ac4C(±) GAPDH mRNA template. (**F**) eGFP expression in RD cells transfected with WT or ac4C mutants (harboring the eGFP reporter vector) at 24 h post-transfection. Scale bars, 50 μm. (**G**) Fluorescence intensity of eGFP in RD cells transfected with WT or ac4C mutants eGFP reporter Vectors at 24 h post-transfection was quantified using ImageJ. Data are means ± SEMs (n = 3). **$P \leq 0.01$, unpaired Student's *t*-tests. (**H**) Binding of PCBP2 and EV71 RNA *in vitro*. EV71 RNAs extracted from T7 transcripts (ac4C[±]) or WT and ac4C mutant virions were incubated with GST-PCBP2. The samples were subjected to IP, followed by quantification

using qRT-PCR. Data are means ± SEMs (*n* = 3). **P* ≤ 0.05, ***P* ≤ 0.01, unpaired Student's *t*-tests. (**I**) Western blotting of extracts from EV71-infected Vero cells treated with shNC or shPCBP2. The expression of PCBP2 was assessed using anti-PCBP2 antibodies, and GAPDH served as a loading control. (**J**) shNC- or shPCBP2-treated Vero cells were infected with EV71 and used to analyze the input RNA and ribosome-loaded RNA levels of *GAPDH* at 24 hpi. Data are means ± SEMs (*n* = 3). ns, not significant, unpaired Student's *t*-tests.

A

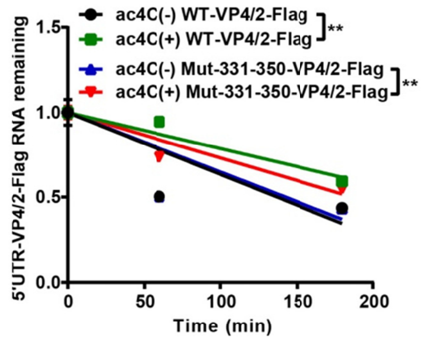

B

**Figure 4B**	WT-3Dmut	Mut-331-3Dmut	Mut-350-3Dmut	Mut-331-350-3Dmut
**Slope±SD**	-0.06601 ± 0.01107	-0.09804 ± 0.01085	-0.1096 ± 0.01014	-0.1238 ± 0.01572
**Figure 4C**	WT-3Dmut	Mut-331-3Dmut	Mut-350-3Dmut	Mut-331-350-3Dmut
**Slope±SD**	-0.03569 ± 0.004698	-0.06140 ± 0.007066	-0.08747 ± 0.008548	-0.1097 ± 0.01269
**Figure 4D**	WT-3Dmut	Mut-331-3Dmut	Mut-350-3Dmut	Mut-331-350-3Dmut
**Slope±SD**	-0.07990 ± 0.01371	-0.09434 ± 0.01348	-0.06618 ± 0.01273	-0.08453 ± 0.01298
**Figure 4E**	WT-3Dmut	WT-3Dmut-ac4C(+)		
**Slope±SD**	-0.07903 ± 0.002439	-0.03428 ± 0.002746		
**Figure 4F**	Mut-331-350-3Dmut	Mut-331-350-3Dmut-ac4C(+)		
**Slope±SD**	-0.1283 ± 0.009602	-0.07993 ± 0.004667		
**Figure 4G**	DMSO-WT	Cycloheximide-WT	DMSO-Mut-331-350-3Dmut	Cycloheximide-Mut-331-350-3Dmut
**Slope±SD**	-0.04908 ± 0.002213	-0.08569 ± 0.01272	-0.07917 ± 0.01131	-0.08417 ± 0.01377
**Figure 4H**	shNC	shPCBP2-1	shPCBP2-2	
**Slope±SD**	-0.06356 ± 0.005627	-0.1019 ± 0.006670	-0.1073 ± 0.008226	
**Figure S4A**	ac4C(-) WT-VP4/2-Flag	ac4C(+) WT-VP4/2-Flag	ac4C(-) Mut-331-350-VP4/2-Flag	ac4C(+) Mut-331-350-VP4/2-Flag
**Slope±SD**	-0.003645 ± 0.0005569	-0.002126 ± 0.0001539	-0.003507 ± 0.0004914	-0.002676 ± 0.0002148

Figure S4. ac4C enhanced the stability of EV71 RNA. (**A**) qRT-PCR was performed to measure the RNA levels of ac4C(±) WT- or Mut-331-350-VP4/2-Flag RNA. Decay graphs were generated by applying linear regression analysis. Data are means ± SEMs (*n* = 3). ***P* ≤ 0.01, two-way ANOVA. (**B**) The slopes of all degradation curves are shown. Data are mean slopes ± SDs.

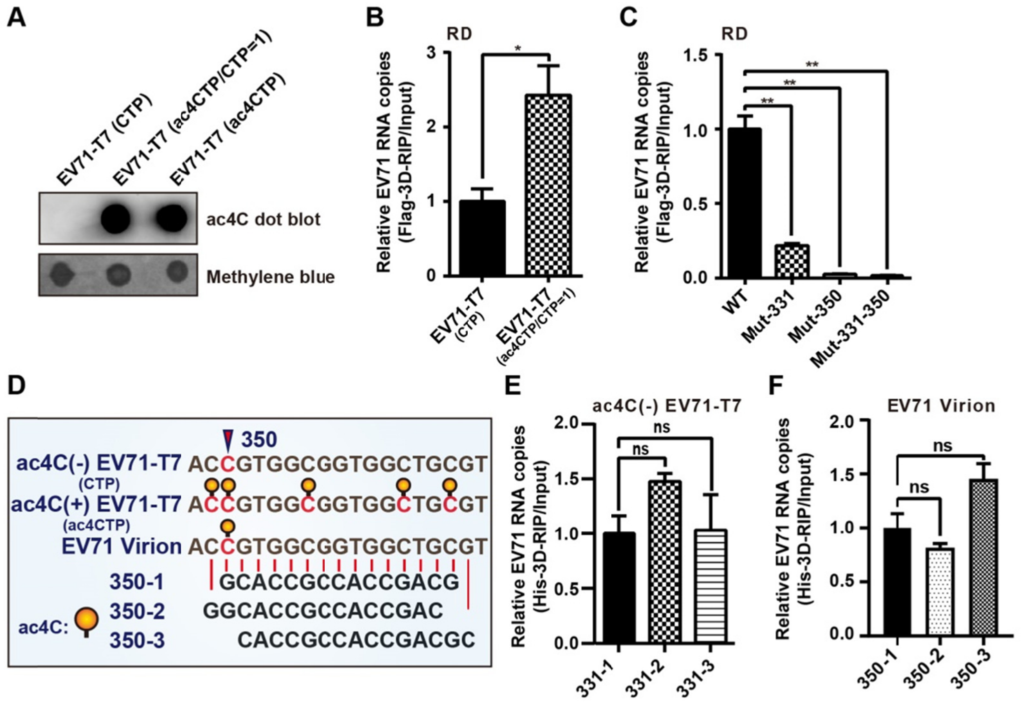

Figure S5. ac4C enhanced the combination of 3D and EV71 RNA. (**A**) Anti-ac4C dot blot was performed on EV71 genome transcribed by T7 with (ac4CTP/CTP=1 and ac4CTP) or without (CTP) ac4C added, with methylene blue staining as loading control. (**B** & **C**) Binding of 3D to EV71 RNA with different ac4C levels in RD cells. Flag-3D overexpressed RD cells were transfected by EV71 genomes with (ac4C/C=50%) or without (ac4C/C=0) ac4C (B) or infected by EV71 WT and ac4C mutants (C), followed by crosslinked-RIP with Flag antibodies and quantified by qRT-PCR. Data are means ± SEMs (*n* = 3). **P* ≤ 0.05, ***P* ≤ 0.01, unpaired Student's *t*-tests. (**D**) Schematic of EV71 RNA pairing with primers near position 350. Yellow solid circles indicate ac4C modification. (**E** & **F**) The 3D binding levels of different 331 primers annealed with ac4C(-) EV71-T7 (E) and that of different 350 primers annealed with EV71 virion RNA (F). Data are means ± SEMs (*n* = 3). ns, not significant, unpaired Student's *t*-tests.

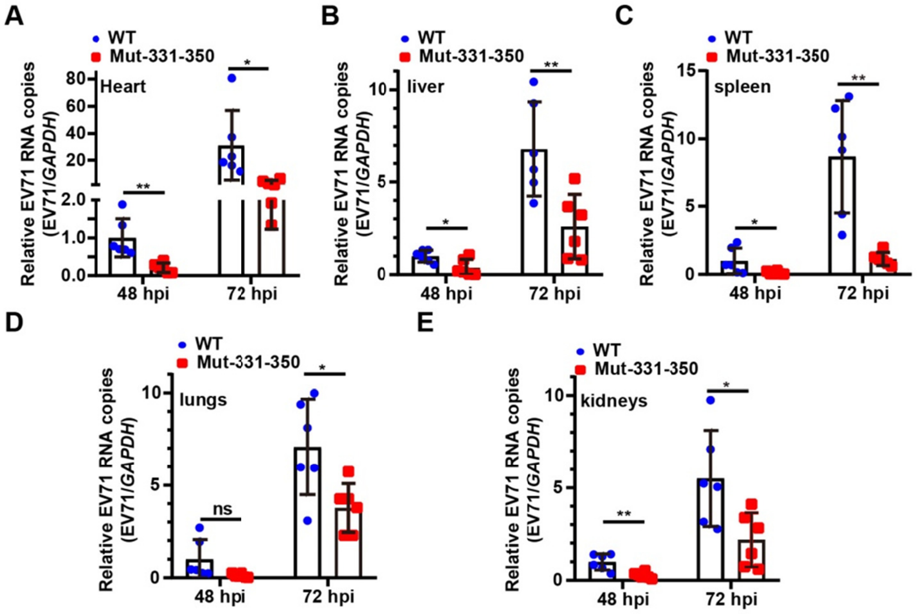

Figure S6. EV71 ac4C mutant caused reduced pathogenicity in mice. (A - E) Viral RNA in organs, including heart (A), liver (B), spleen (C), lungs (D) and kidneys (E), from AG6 mice infected with EV71 were quantified by qPCR. Data are means ± SEMs (n = 6). *$P \leq 0.05$, **$P \leq 0.01$, ns: not significant, unpaired Student's *t*-tests.

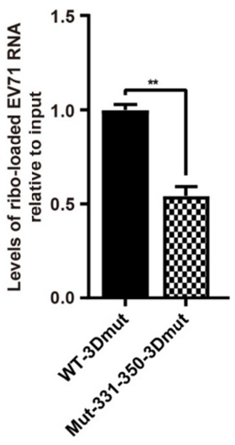

Figure S7. The effect of ac4C on EV71 translation was independent of 3D. WT-3Dmut- or Mut-331-350-3Dmut-transfected-Vero cells were used to analyze the input RNA and ribosome loaded RNA levels of EV71 at 8 h post transfection. Data are means ± SEMs (*n* = 3). $^{**}P \leq 0.01$, unpaired Student's *t*-tests.
